# Dietary sugar consumption and health: umbrella review

**DOI:** 10.1136/bmj-2022-071609

**Published:** 2023-04-05

**Authors:** Yin Huang, Zeyu Chen, Bo Chen, Jinze Li, Xiang Yuan, Jin Li, Wen Wang, Tingting Dai, Hongying Chen, Yan Wang, Ruyi Wang, Puze Wang, Jianbing Guo, Qiang Dong, Chengfei Liu, Qiang Wei, Dehong Cao, Liangren Liu

**Affiliations:** 1 Department of Urology/Institute of Urology, West China Hospital, Sichuan University, Chengdu, China; 2 Department of Oncology, West China Hospital, Sichuan University, Chengdu, China; 3 Chinese Evidence-based Medicine Center, West China Hospital, Sichuan University, Chengdu, China; 4 Department of Clinical Nutrition, West China Hospital, Sichuan University, Chengdu, China; 5 Research Core Facility, West China Hospital, Sichuan University, Chengdu, China; 6 Department of Urologic Surgery, UC Davis School of Medicine, Sacramento, CA, USA

## Abstract

**Objective:**

To evaluate the quality of evidence, potential biases, and validity of all available studies on dietary sugar consumption and health outcomes.

**Design:**

Umbrella review of existing meta-analyses.

**Data sources:**

PubMed, Embase, Web of Science, Cochrane Database of Systematic Reviews, and hand searching of reference lists.

**Inclusion criteria:**

Systematic reviews and meta-analyses of randomised controlled trials, cohort studies, case-control studies, or cross sectional studies that evaluated the effect of dietary sugar consumption on any health outcomes in humans free from acute or chronic diseases.

**Results:**

The search identified 73 meta-analyses and 83 health outcomes from 8601 unique articles, including 74 unique outcomes in meta-analyses of observational studies and nine unique outcomes in meta-analyses of randomised controlled trials. Significant harmful associations between dietary sugar consumption and 18 endocrine/metabolic outcomes, 10 cardiovascular outcomes, seven cancer outcomes, and 10 other outcomes (neuropsychiatric, dental, hepatic, osteal, and allergic) were detected. Moderate quality evidence suggested that the highest versus lowest dietary sugar consumption was associated with increased body weight (sugar sweetened beverages) (class IV evidence) and ectopic fatty accumulation (added sugars) (class IV evidence). Low quality evidence indicated that each serving/week increment of sugar sweetened beverage consumption was associated with a 4% higher risk of gout (class III evidence) and each 250 mL/day increment of sugar sweetened beverage consumption was associated with a 17% and 4% higher risk of coronary heart disease (class II evidence) and all cause mortality (class III evidence), respectively. In addition, low quality evidence suggested that every 25 g/day increment of fructose consumption was associated with a 22% higher risk of pancreatic cancer (class III evidence).

**Conclusions:**

High dietary sugar consumption is generally more harmful than beneficial for health, especially in cardiometabolic disease. Reducing the consumption of free sugars or added sugars to below 25 g/day (approximately 6 teaspoons/day) and limiting the consumption of sugar sweetened beverages to less than one serving/week (approximately 200-355 mL/week) are recommended to reduce the adverse effect of sugars on health.

**Systematic review registration:**

PROSPERO CRD42022300982.

## Introduction

As an important component of the human diet, sugars have been shown to be harmfully associated with a variety of risk factors for decades, mainly including obesity,^1^ ^2^ ^3^ diabetes,^4^ ^5^ ^6^ cardiovascular disease,^7^ ^8^ ^9^ ^10^ hyperuricaemia,^11^ gout,^11^ ^12^ ^13^ ectopic fatty accumulation,^14^ ^15^ ^16^ dental caries,^17^ and some cancers.^18^ ^19^ ^20^ ^21^ According to the latest report of the World Health Organization and the Food and Agriculture Organization of the United Nations, sugars include monosaccharides, disaccharides, polyols, and free sugars, of which free sugars are identified as all monosaccharides and disaccharides added to foods by the manufacturer, cook, or consumer and sugars naturally present in honey, syrups, and fruit juices.^3^ ^22^ In addition, another important group of sugars, added sugars, has been proposed in the Dietary Guidelines for Americans and has been defined as all monosaccharides and disaccharides used in processed and prepared foods and drinks and sugars added to foods but not naturally occurring sugars such as in fruits and fruit juices (table 1).^23^

**Table 1:** Classification of dietary sugars^3^ ^23^

Class	Principal components
Monosaccharides	Glucose, fructose, galactose
Disaccharides	Sucrose, lactose, maltose, trehalose
Polyols	Sorbitol, mannitol, lactitol, xylitol, erythritol, isomalt, maltitol
Free sugars	All monosaccharides and disaccharides added to foods by the manufacturer, cook, or consumer; sugars naturally present in honey, syrups, and fruit juices
Added sugars	All monosaccharides and disaccharides used in processed and prepared foods and drinks; sugars added to foods but not naturally occurring sugars such as in fruits and fruit juices

In recent years, many studies have focused on the adverse effects of sugar sweetened beverages on human health, given the substantial contribution of these drinks to total added sugar or free sugar intake and the rapidly increasing rate of their consumption.^24^ ^25^ ^26^ Generally, sugar sweetened beverages are the largest source of added sugars, including carbonated and noncarbonated soft drinks, fruit drinks, and sports and energy drinks.^27^ Previous surveys have shown that consumption of sugar sweetened beverages is declining in many developed countries, although consumption levels remain high.^27^ ^28^ However, the consumption of sugar sweetened beverages is still increasing in many developing countries, which may be attributed to their increased availability accompanied by economic development.^29^ The 2007 annual report of the Coca-Cola company revealed that the consumption of sugar sweetened beverages in India and China increased by 14% and 18%, respectively, in one year.^30^ In 2018 a cross sectional survey conducted among Chinese primary and junior high school students showed that sugar sweetened beverages provide 10-15% of the total calorie consumption of school students.^31^ Data from the National Health and Nutrition Examination Survey (NHANES) showed that, in 2009-10, sugar sweetened beverage consumption contributed 8% and 6.9% of daily energy intake among children/adolescents and adults, respectively, in the US.^32^ Additionally, a global survey conducted in 2010 reported that a total of 180 000 adiposity associated deaths could be attributed to the consumption of sugar sweetened beverages around the world.^33^ All of these findings promote the development of policies worldwide to limit sugar consumption, including sugars taxes, food labelling laws, and restrictions on advertising and marketing.^34^ ^35^ ^36^ ^37^ Meanwhile, national and international organisations such as WHO, the US Department of Agriculture, and the US Department of Health and Human Services have recommended reducing the consumption of free sugars or added sugars to less than 10% of total daily energy intake.^23^ ^38^

Although many meta-analyses of observational studies and randomised controlled trials focused on the associations between sugar consumption and a range of health outcomes have been published in recent decades, deficiencies in the study design, varying measurements of dietary sugar consumption, inconsistent findings, and different definitions of exposure make drawing definitive conclusions difficult. Therefore, before developing detailed policies for sugar restriction, the quality of existing evidence on the associations of dietary sugar consumption with all health outcomes needs to be comprehensively evaluated. To evaluate the quality of evidence, potential biases, and validity of all studies available on dietary sugar intake and any health outcomes, we did an umbrella review of meta-analyses on this topic.

## Methods

### Umbrella review methods

We systematically searched, extracted, and analysed large amounts of data from published systematic reviews and meta-analyses that research the associations between various health outcomes and dietary sugar consumption.^39^ ^40^ Generally, dietary sugar consumption could be measured through the specific proportions of sugars in foods or a percentage of total energy and combined in meta-analyses.^3^ Therefore, we excluded simple systematic reviews without meta-analyses from our umbrella review. We prospectively registered this umbrella review in PROSPERO (CRD42022300982) (https://www.crd.york.ac.uk/PROSPERO/).

### Literature search

We searched PubMed, Embase, Web of Science, and the Cochrane Database of Systematic Reviews from inception through January 2022 (last update) for systematic reviews and meta-analyses of randomised controlled trials and observational studies. We searched the databases through a combination of Medical Subject Headings terms, keywords, and variations of text words associated with sugars following the Scottish Intercollegiate Guidelines Network’s guidance for literature searching: (sugars OR sugar) AND (systematic review OR meta-analysis).^41^ Two authors (YH and ZYC) separately conducted electronic searches to screen the titles and abstracts retrieved from the databases and identified meta-analyses that met the inclusion criteria by full text reading. Any discrepancy in the literature screening between the two reviewers was resolved by a third author (LRL). We hand searched meta-analyses and reviews from the reference lists of all included articles to identify studies that might have been missed.

### Eligibility criteria

We identified dietary sugar consumption as the intake of total sugars and the consumption of a component of total sugars (monosaccharides, disaccharides, polyols, free sugars, or added sugars), which are expressed in absolute amounts or as a percentage of total energy, or the intake of sugar sweetened beverages or foods (table 1).^3^ We included systematic reviews and meta-analyses of randomised controlled trials, cohort studies, case-control studies, or cross sectional studies that evaluated dietary sugar consumption in humans free from acute or chronic diseases. Meta-analyses were eligible for inclusion when they compared the effects of different dietary sugar consumption on the same health outcome through relative risks, odds ratios, hazard ratios, weighted mean differences, or standardised mean differences. We included meta-analyses when the exposure was total sugars, monosaccharides, disaccharides, polyols, free sugars, added sugars, or sugar sweetened beverages or foods. We extracted data on individual outcomes separately if two or more health outcomes were reported in a study. If more than one study published more than 24 months apart was conducted on the same dietary sugar exposure and health outcomes, we included the most recent study for data extraction, which is generally the study with the largest sample size. If more than one study was conducted within the same 24 month period, we included the meta-analysis with the largest number of prospective cohort studies and randomised controlled trials (a study with a higher AMSTAR score was included if the number of prospective studies was equal).^42^ ^43^ Furthermore, if the most recent study did not do dose-response analysis, whereas another study did, we included both studies for data extraction.

The exclusion criteria for these umbrella reviews included meta-analyses of the association between carbohydrates, non-nutritive sweeteners, and artificially sweetened beverages and health outcomes; meta-analyses evaluating the therapeutic or metabolic effects of short term sugar supplementation; meta-analyses that evaluated the effects of dietary sugar consumption on health outcomes in certain disease populations; randomised controlled trials that aimed to achieve isoenergetic replacement of sugars with other forms of carbohydrate; studies with insufficient data to evaluate sugar consumption from sugar containing foods (such as honey, apples, chocolate, ice cream, 100% fruit juice); and non-English studies and animal and cell culture studies.

### Data extraction

Two reviewers (YH and ZYC) independently extracted the following information from each eligible study: first author’s name, publication year, type of dietary sugar consumption (total sugars, monosaccharides, disaccharides, polyols, free sugars, added sugars, sugar sweetened beverages or foods), measurement of dietary sugar consumption, health outcome, number of included studies, number of cases and total participants, study design (cross sectional, case-control, cohort, and randomised controlled trial), comparison (high versus low, never/low versus moderate/high, any versus none, or extra increment of sugars per day (or week) versus none), and estimated summary effect (risk ratio, odds ratio, weighted mean difference, and standardised mean difference with 95% confidence intervals). Furthermore, we extracted the model of effect (random and fixed), heterogeneity (I^2^ statistic and Cochran’s Q test P value), and publication bias assessment (P value of Egger’s test or funnel plot). If dose-response analysis and subgroup analysis were conducted, we also extracted the non-linearity tests’ P value and results of subgroup analysis in meta-analyses. If a meta-analysis was conducted on both cohort and case-control/cross sectional studies and stratification analysis was conducted through study design, we selected the cohort design subanalysis results for data extraction or reanalysed. Any disagreement was determined by a third author (LRL).

### Quality assessment of methods and evidence

Two reviewers (YH and ZYC) evaluated the methodological quality of the included articles by using AMSTAR (a measurement tool to assess systematic reviews), a valid and dependable measurement tool in assessing the quality of systematic reviews and meta-analyses.^42^ ^44^ In addition, according to the Grading of Recommendations, Assessment, Development and Evaluation (GRADE), we evaluated evidence of each health outcome and graded it as “high,” “moderate,” “low,” or “very low” quality to draw conclusions.^45^ Additionally, we classified evidence of outcomes into four categories following the evidence classification criteria: class I (convincing evidence), class II (highly suggestive evidence), class III (suggestive evidence), class IV (weak evidence), and NS (non-significant).^46^ ^47^ ^48^ Table 2 shows detailed criteria of evidence classification.

**Table 2:** Evidence classification criteria^46^ ^47^ ^48^

Evidence class	Description
Class I: convincing evidence	>1000 cases (or >20 000 participants for continuous outcomes); statistical significance at P<10^−6^ (random effects); no evidence of small study effects and excess significance bias; 95% prediction interval excluded null value; no large heterogeneity (I^2^<50%)
Class II: highly suggestive evidence	>1000 cases (or >20 000 participants for continuous outcomes), statistical significance at P<10^−6^ (random effects), and largest study with 95% confidence interval excluding null value
Class III: suggestive evidence	>1000 cases (or >20 000 participants for continuous outcomes) and statistical significance at P<0.001
Class IV: weak evidence	Remaining significant associations with P<0.05
NS: non-significant	P>0.05

### Data analysis

We reanalysed the risk ratio, odds ratio, weighted mean difference, or standardised mean difference with 95% confidence intervals through random or fixed effects models and calculated the I^2^ statistic, P value of Cochran’s Q test for heterogeneity, and P value of Egger’s regression test (at least 10 studies were included) for small study effects in each included meta-analysis that reported the metric, number of cases, and participants of the included original studies.^49^ ^50^ ^51^ For outcomes classified as class I or II, we did sensitivity analysis if sufficient data were available to assess whether the credibility of the evidence varied when some component studies were excluded. We also extracted dose-response associations between dietary sugar consumption and various health outcomes from the included meta-analyses, if available. Moreover, if the latest meta-analysis did not include the original articles that were included by other meta-analyses, we combined the data of these meta-analyses and did a reanalysis. We assessed agreement statistics between two authors (YH and ZYC) regarding study selection by using Cohen’s κ statistics and associated 95% confidence interval. We interpreted magnitude of agreement by following guidelines reported by Landis and Koch: slight (0.00-0.20), fair (0.21-0.40), moderate (0.41-0.60), substantial (0.61-0.80), and almost perfect agreement (0.81-1.00).^52^ In addition, if a meta-analysis reported the estimated effect by combining observational studies with randomised controlled trials, we reanalysed the estimated effects for observational studies and randomised controlled trials separately. If we could not do a reanalysis from a meta-analysis, we extracted summary data and assessed heterogeneity and publication bias from the meta-analysis as far as possible. We identified a P value <0.10 as statistically significant for heterogeneity tests. For other tests, we considered a P value <0.05 to be significant. We used Review Manager version 5.3 for evidence synthesis, Stata version 12.1 for Egger’s test and sensitivity analysis, and IBM SPSS Statistics version 25 for Cohen’s κ statistics.

### Patient and public involvement

Patients and the public were not involved in the planning, design, and implementation of the study, as this study used secondary data. No patients were asked to advise on interpretation or writing up of the manuscript.

## Results

### Characteristics of meta-analyses

**Fig 1:**
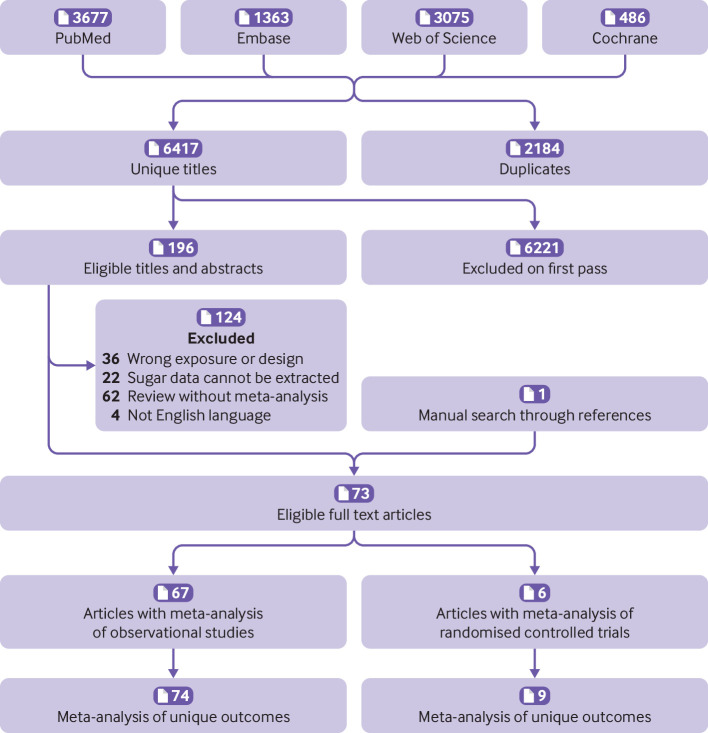
Flowchart of systematic search and selection process

**Fig 2:**
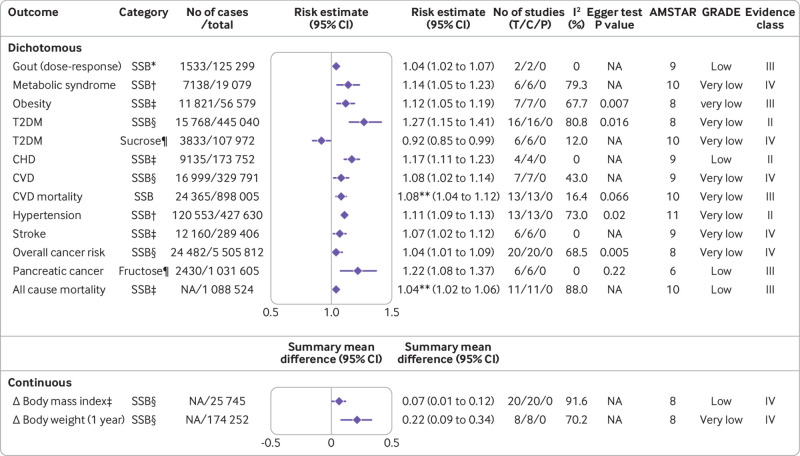
Significant dose-response relations between dietary sugar consumption and multiple health outcomes. Estimates are relative risks, summary mean difference is weighted mean difference, and effect models are random unless noted otherwise. Δ=final value – baseline value; AMSTAR=a measurement tool to assess systematic reviews; C=cohort studies; CHD=coronary heart disease; CI=confidence interval; CVD=cardiovascular disease; GRADE=Grading of Recommendations Assessment, Development and Evaluation; NA=not available; P=population based case-control and/or cross sectional studies; SSB=sugar sweetened beverage; T=total No of studies; T2DM_=_type 2 diabetes mellitus. *1 serving/week increment. †355 mL/d increment. ‡250 mL/d increment. §1 serving/d increment. ¶25 g/d increment. **Hazard ratio. †Children

**Fig 3:**
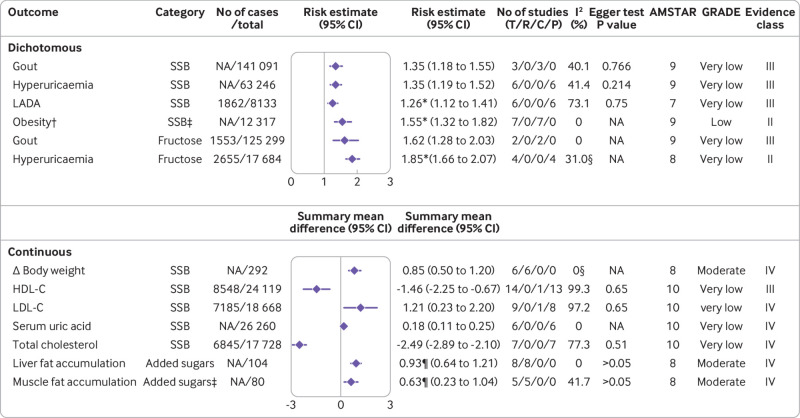
Significant non-dose-response relations between dietary sugar consumption and endocrine and metabolic outcomes. Comparisons are highest versus lowest, estimates are relative risks, summary mean difference is weighted mean difference, and effect models are random unless noted otherwise. Complete associations between dietary sugar consumption and endocrine and metabolic outcomes are shown in supplementary table A. Δ=final value – baseline value; AMSTAR=a measurement tool to assess systematic reviews; C=cohort studies; CI=confidence interval; GRADE=Grading of Recommendations Assessment, Development and Evaluation; HDL-C=high density lipoprotein cholesterol; LADA=latent autoimmune diabetes in adults; LDL-C=low density lipoprotein cholesterol; NA=not available; P=population based case-control and/or cross sectional studies; R=randomised controlled trials; SSB=sugar sweetened beverage; T=total No of studies. *Odds ratio. †Children. ‡Any versus none. §Fixed effects model. ¶Standardised mean difference

**Fig 4:**
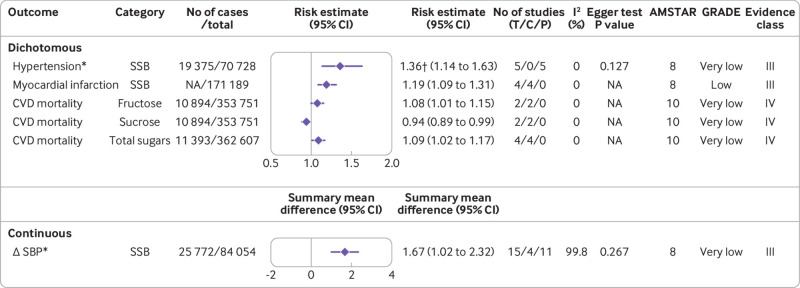
Significant non-dose-response relations between dietary sugar consumption and cardiovascular outcomes. Comparisons are highest versus lowest, estimates are relative risks, summary mean difference is weighted mean difference, and effect models are random unless noted otherwise. Complete associations between dietary sugar consumption and cardiovascular outcomes are shown in supplementary table B. Δ=final value – baseline value; AMSTAR=a measurement tool to assess systematic reviews; C=cohort studies; CI=confidence interval; CVD=cardiovascular disease; GRADE=Grading of Recommendations Assessment, Development and Evaluation; NA=not available; P=population based case-control and/or cross sectional studies; SBP=systolic blood pressure; SSB=sugar sweetened beverage; T=total No of studies. *Children and adolescents. †Odds ratio

**Fig 5:**
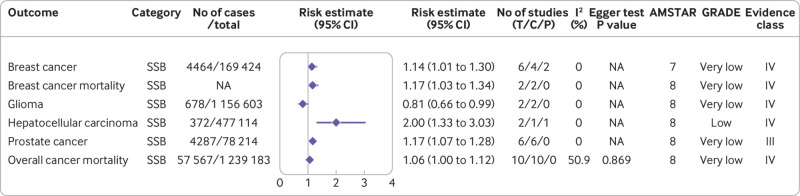
Significant non-dose-response relations between dietary sugar consumption and cancer outcomes. Comparisons are highest versus lowest, estimates are relative risks, and effect models are random unless noted otherwise. Complete associations between dietary sugar consumption and cancer outcomes are shown in supplementary table C. AMSTAR=a measurement tool to assess systematic reviews; GRADE=Grading of Recommendations Assessment, Development and Evaluation; C=cohort studies; CI=confidence interval; NA=not available; P=population based case-control and/or cross sectional studies; SSB=sugar-sweetened beverage; T=total No of studies

**Fig 6:**
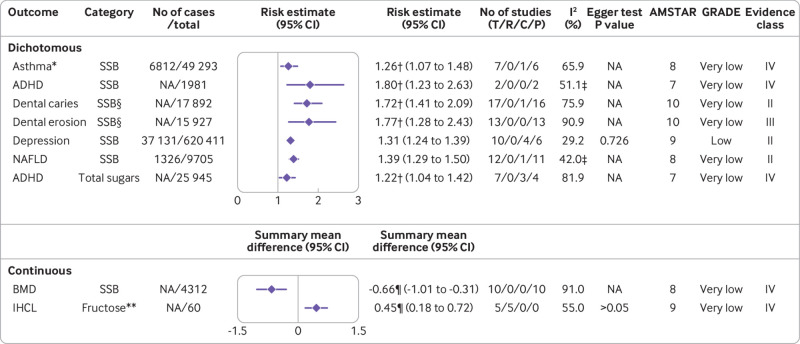
Significant non-dose-response relations between dietary sugar consumption and other outcomes. Comparisons are highest versus lowest, estimates are relative risks, summary mean difference is weighted mean difference, and effect models are random unless noted otherwise. Complete associations between dietary sugar consumption and other outcomes are shown in supplementary table D. ADHD=attention deficit/hyperactivity disorder; AMSTAR=a measurement tool to assess systematic reviews; BMD=bone mineral density; C=cohort studies; CI=confidence interval; GRADE=Grading of Recommendations Assessment, Development and Evaluation; IHCL=intrahepatocellular lipids; NA=not available; NAFLD=non-alcoholic fatty liver disease; P=population based case-control and/or cross sectional studies; R=randomised controlled trials; SSB=sugar-sweetened beverage; T=total No of studies. *Children. †Odds ratio. ‡Fixed effects model. §Never/low versus moderate/high consumption. ¶Standardised mean difference. **Any versus none

Figure 1 shows the flowchart of the literature search and selection process. After a systematic literature search, we identified 8601 unique articles. Application of our inclusion criteria yielded total of 73 meta-analyses, including 67 meta-analyses of observational studies and six meta-analyses of randomised controlled trials. Agreement between the two reviewers (YH and ZYC) for study selection was almost perfect (κ=0.906, 95% confidence interval 0.859 to 0.953; P<0.001). We extracted 74 unique outcomes in meta-analyses of observational studies and nine unique outcomes in meta-analyses of randomised controlled trials. Meta-analyses of randomised controlled trials included only change in body weight (sugar sweetened beverages), liver fat accumulation, muscle fat accumulation, change in body mass index, change in body weight (fructose), postprandial triglycerides, serum uric acid, intrahepatocellular lipids, and alanine aminotransferase. Figure 2 shows the significant dose-response relations between dietary sugar consumption and multiple health outcomes. The other forest plots show the significant non-dose-response relations between dietary sugar consumption and endocrine/metabolic (fig 3), cardiovascular (fig 4), cancer (fig 5), and other outcomes (fig 6). The full versions of the associations between dietary sugar consumption and each outcome are shown in supplementary tables A-D.

**Fig 7:**
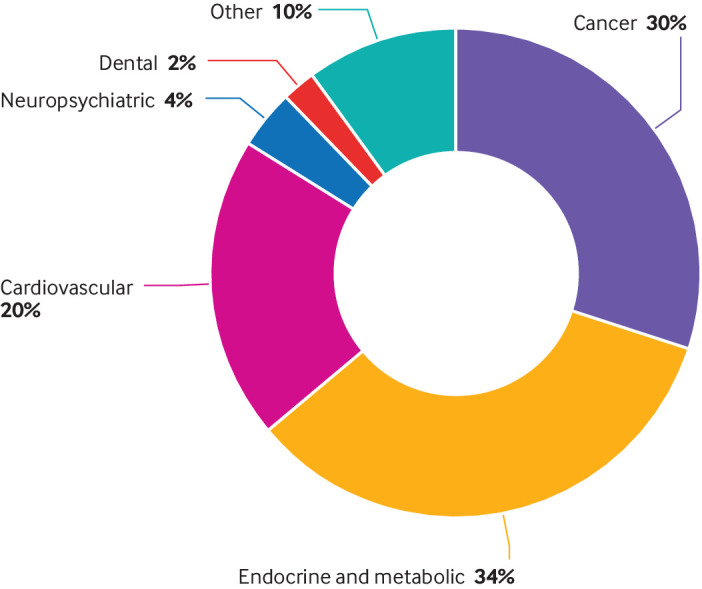
Map of outcomes associated with dietary sugar consumption

Most of the included meta-analyses focused on the associations between dietary sugar consumption and endocrine/metabolic diseases (n=28), followed by cancer (n=25), cardiovascular diseases (n=17), neuropsychiatric diseases (n=3), dental diseases (n=2), and other diseases (n=8) (fig 7). Dietary sugar exposure included sugar sweetened beverages (n=58), fructose (n=11), sucrose (n=4), lactose (n=1), added sugars (n=4), free sugars (n=1), and total sugars (n=4). Significance was reached for 45 harmful associations and four beneficial associations. The remaining 34 outcomes were either harmfully or beneficially associated but did not reach significance. After quality assessment of evidence through GRADE and evidence classification criteria, most of the 83 outcomes were classified as “low” or “very low” quality and III, IV, or NS evidence class. Only four (5%) endocrine/metabolic outcomes were classified as “moderate” quality. Three (4%) endocrine/metabolic outcomes, two (2%) cardiovascular outcomes, and three (4%) other outcomes were graded as class IIB. No “high” quality or class I evidence was found in this umbrella review.

### Endocrine and metabolic outcomes

#### Low and moderate quality evidence

A meta-analysis of six randomised controlled trials found that sugar sweetened beverage consumption was significantly associated with increased body weight for highest versus lowest consumption (weighted mean difference 0.85, 95% confidence interval 0.50 to 1.20) (moderate; IV (the quality of evidence is expressed as “GRADE, evidence class”)).^53^ In addition, any versus no added sugar consumption was associated with increased liver fat accumulation (standardised mean difference 0.93, 95% confidence interval 0.64 to 1.21) (moderate; IV) and muscle fat accumulation (standardised mean difference 0.63, 0.23 to 1.04) (moderate; IV).^54^ Another dose-response meta-analysis showed that a one serving/week increment in artificially sweetened beverages was associated with a 4% higher risk of gout (risk ratio 1.04, 95% confidence interval 1.02 to 1.07) (low; III).^13^ Furthermore, comparison of higher sugar sweetened beverage consumption with non-sugar sweetened beverage consumption indicated a 55% (odds ratio 1.55, 95% confidence interval 1.32 to 1.82) increased risk of obesity in children associated with higher consumption (low; II).^3^ Sugar sweetened beverage consumption was also linked with an increased body mass index in children.^53^ The authors conducted a dose-response analysis and showed that body mass index in children increased by 0.07 units for every one serving/day increment of sugar sweetened beverages (weighted mean difference 0.07, 0.01 to 0.12) (low; IV).^53^ Evidence from this umbrella review suggests that fructose intake was not associated with serum uric acid (moderate; NS)^55^ or changes in body weight (low; NS) (fig 2; fig 3).^56^

#### Very low quality evidence

Dose-response analysis based on seven cohort studies showed that a one serving/day increment of sugar sweetened beverages was associated with a 0.22 kg weight gain in one year (weighted mean difference 0.22, 0.09 to 0.34).^53^ Furthermore, the risk of gout increased by 35% (risk ratio 1.35, 1.18 to 1.55) for the highest versus lowest sugar sweetened beverage consumption.^11^ The highest versus lowest sugar sweetened beverage consumption was also significantly associated with a 35% (risk ratio 1.35, 1.19 to 1.52) higher risk of hyperuricaemia.^11^ In addition, another pooled analysis found that participants with the highest sugar sweetened beverage consumption had 0.18 mg/dL greater concentrations of serum uric acid than did those with the lowest consumption (weighted mean difference 0.18, 0.11 to 0.25).^57^ Similarly, the highest fructose intake could also increase the risk of gout (risk ratio 1.62, 1.28 to 2.03)^58^ and hyperuricaemia (odds ratio 1.85, 1.66 to 2.07)^59^ compared with the lowest consumption.

The most recent meta-analysis found a 1.46 mg/dL (weighted mean difference −1.46, −2.25 to −0.67) decrement of high density lipoprotein cholesterol for the highest versus lowest sugar sweetened beverage consumption.^60^ Subgroup analysis indicated that the highest versus lowest sugar sweetened beverage consumption was associated with lower high density lipoprotein cholesterol in studies conducted in the US (weighted mean difference −2.85, −4.09 to −1.61) but was associated with higher high density lipoprotein cholesterol in studies conducted in Europe/Oceania (weighted mean difference 1.65, 0.26 to 3.05).^60^ The highest versus lowest sugar sweetened beverage consumption was also significantly associated with increased low density lipoprotein cholesterol (weighted mean difference 1.21, 0.23 to 2.20) and decreased total cholesterol (−2.49, −2.89 to −2.10).^60^ After stratification by region, no significant association between sugar sweetened beverage consumption and low density lipoprotein cholesterol was detected in the US, Europe/Oceania, and Asia,^60^ whereas the highest versus lowest sugar sweetened beverage consumption was associated with lower total cholesterol concentrations in studies conducted in the US/Europe (weighted mean difference −2.47, −2.88 to −2.07) but not in Asia.^60^

The risk of metabolic syndrome was increased by 14% (risk ratio 1.14, 1.05 to 1.23) for a 355 mL/day increment of sugar sweetened beverages, with no evidence for departure from linearity.^61^ In addition, a meta-analysis including 56 579 participants and 11 821 incident cases of obesity showed an adverse linear dose-response association between sugar sweetened beverage consumption and the risk of obesity.^1^ Each 250 mL/day increment in sugar sweetened beverage consumption was associated with a 12% (risk ratio 1.12, 1.05 to 1.19) higher risk of obesity, and this association also remained after adjustment for energy intake (1.13, 1.09 to 1.18) and physical activity (1.14, 1.05 to 1.25).^1^ Moreover, a meta-analysis of 16 cohort studies found that with each one serving/day increment of sugar sweetened beverage consumption, the risk of developing type 2 diabetes mellitus increased by 27% (risk ratio 1.27, 1.15 to 1.41).^6^ By contrast, an 8% (risk ratio 0.92, 0.85 to 0.99) lower risk of type 2 diabetes mellitus for each 25 g/day increment in sucrose intake was confirmed in dose-response analysis based on six cohort studies.^62^ The highest versus lowest sugar sweetened beverage consumption was also significantly associated with a higher risk of latent autoimmune diabetes in adults (odds ratio 1.26, 1.12 to 1.41) (fig 2; fig3).^30^

We found no significant association between sugar sweetened beverage consumption and changes in body mass index in adults,^63^ triglycerides,^60^ or large waist circumference.^64^ Fructose intake was not associated with postprandial triglycerides or type 2 diabetes mellitus.^62^ ^65^ Total sugar consumption was also not associated with type 2 diabetes mellitus (supplementary table A).^62^

### Cardiovascular outcomes

#### Low quality evidence

In a single article,^10^ a positive association between sugar sweetened beverage consumption and the risk of coronary heart disease was observed. Dose-response analysis showed that each 250 mL/day increment of sugar sweetened beverage consumption was positively associated with a 17% (risk ratio 1.17, 1.11 to 1.23) higher risk of coronary heart disease (low; II).^10^ In addition, extreme category analysis showed that the highest versus lowest sugar sweetened beverage consumption was associated with an increased risk of myocardial infarction (risk ratio 1.19, 1.09 to 1.31) (low; III).^66^ Low quality evidence suggests that fructose intake was not associated with the risk of hypertension (low; NS) (fig 2; fig 4).^67^

#### Very low quality evidence

Except for a beneficial association between sucrose intake and cardiovascular disease mortality, all categories of dietary sugar exposure were adversely associated with various cardiovascular outcomes. A recent dose-response meta-analysis showed that each 250 mL/day increment of sugar sweetened beverage consumption was positively associated with a 7% (risk ratio 1.07, 1.02 to 1.12) higher risk of stroke.^10^ Another meta-analysis of seven cohort studies with 329 791 participants and 16 999 cases found that each one serving/day increment of sugar sweetened beverage consumption was linearly associated with an 8% (risk ratio 1.08, 1.02 to 1.14) increased risk of cardiovascular disease.^8^ For cardiovascular disease mortality, each serving/day increment of sugar sweetened beverage consumption was also linearly associated with a higher risk (hazard ratio 1.08, 1.04 to 1.12).^68^ However, subgroup analysis found that the association between sugar sweetened beverage consumption and cardiovascular disease mortality was not statistically significant among participants from Asia.^68^ In a separate meta-analysis in children and adolescents,^69^ the highest versus lowest sugar sweetened beverage consumption was shown to be associated with a 1.67 mm Hg (weighted mean difference 1.67, 1.02 to 2.32) increase in systolic blood pressure and a 36% (odds ratio 1.36, 1.14 to 1.63) higher risk of hypertension. In adults, the results from pooled analysis of 13 prospective cohort studies indicated a harmful dose-response association between sugar sweetened beverage consumption and incidence of hypertension.^70^ The risk of hypertension was increased by 11% (risk ratio 1.11, 1.09 to 1.13) for a 355 mL/day (1 serving/day) increment in sugar sweetened beverage consumption.^70^ Moreover, both fructose (risk ratio 1.08, 1.01 to 1.15) and total sugars (risk ratio 1.09, 1.02 to 1.17) were harmfully associated with the risk of cardiovascular disease mortality for highest versus lowest consumption,^71^ whereas a beneficial association between sucrose intake and cardiovascular disease mortality was observed (fig 2; fig 4).^71^

We observed no significant association between sugar sweetened beverage consumption and changes in diastolic blood pressure (children and adolescents)^69^ or heart failure.^10^ We also observed no significant association between sucrose intake or total sugar consumption and the risk of cardiovascular disease.^71^ In addition, added sugar consumption was not associated with the risk of cardiovascular disease mortality (supplementary table B).^71^

### Cancer

#### Low quality evidence

A dose-response meta-analysis showed that the risk of hepatocellular carcinoma increased by 100% (risk ratio 2.00, 1.33 to 3.03) for the highest sugar sweetened beverage consumption compared with the lowest (low; IV).^18^ Additionally, a meta-analysis conducted by Aune and colleagues found that 25 g/day of fructose intake was linearly associated with a 22% higher risk of pancreatic cancer (risk ratio 1.22, 1.08 to 1.37) (low; III).^72^ The association between fructose intake and incidence of pancreatic cancer remained significant in the subgroups of studies that adjusted for smoking, body mass index, red and processed meat consumption, and energy intake, whereas the association was diminished in the subgroups of studies that adjusted for alcohol consumption, diabetes status, or physical activity (fig 2; fig 5).^72^

#### Very low quality evidence

A recent meta-analysis of six observational studies showed a higher risk of breast cancer for highest versus lowest sugar sweetened beverage consumption (risk ratio 1.14, 1.01 to 1.30).^19^ In a separate meta-analysis, Li and colleagues found that the highest sugar sweetened beverage consumption might increase the risk of breast cancer mortality by 17% (risk ratio 1.17, 1.03 to 1.34) compared with the lowest.^18^ Moreover, a meta-analysis of six cohort studies showed that participants with the highest sugar sweetened beverage consumption had a higher risk of prostate cancer than those with the lowest intake (risk ratio 1.17, 1.07 to 1.28). Dose-response analysis did not detect a significant association.^18^ However, we observed a protective association between sugar sweetened beverage consumption and glioma in our umbrella review (risk ratio 0.81, 0.66 to 0.99).^18^ In addition, a meta-analysis including 20 cohort studies with 5 505 812 participants observed a positive linear dose-response relation between sugar sweetened beverage consumption and overall cancer risk.^18^ The risk increased by 4% for every serving/day increment of sugar sweetened beverage consumption (risk ratio 1.04, 1.01 to 1.09).^18^ Furthermore, pooled analysis of 10 cohort studies with 1 239 183 participants found that the highest versus lowest sugar sweetened beverage consumption was significantly associated with a higher risk of overall cancer mortality (risk ratio 1.06, 1.00 to 1.12), without a significant dose-response relation.^18^ Stratification by region produced a positive association between sugar sweetened beverage consumption and overall cancer mortality in the North American population (odds ratio 1.08, 1.01 to 1.15) but not in Asia (0.99, 0.81 to 1.22) (fig 2; fig 5).^18^

We observed no significant association between sugar sweetened beverage consumption and the risk of biliary track cancer,^18^ bladder cancer,^18^ colon cancer,^73^ colorectal cancer,^18^ colorectal cancer mortality,^18^ endometrial cancer,^18^ oesophageal cancer,^18^ gastric cancer,^18^ haematological malignancy,^18^ kidney cancer,^18^ lung cancer mortality,^18^ nasopharyngeal carcinoma,^18^ pancreatic cancer,^18^ and prostate cancer mortality.^18^ In addition, added sugar consumption was not associated with the risk of colorectal cancer.^74^ We observed no significant association between sucrose intake and pancreatic cancer.^72^ Moreover, lactose intake was not associated with the risk of ovarian cancer (supplementary table C).^75^

### Other outcomes

#### Low quality evidence

A recent meta-analysis of 11 cohort studies suggested that an increment in sugar sweetened beverage consumption of 250 mL/day was associated with a 4% (hazard ratio 1.04, 1.02 to 1.06) higher risk of all cause mortality (low; III).^76^ Moreover, a harmful association between sugar sweetened beverage consumption and the risk of depression was observed in a meta-analysis of 10 observational studies (risk ratio 1.31, 1.24 to 1.39) (low; II).^77^ No significant association was observed between fructose intake and alanine transaminase concentration (low; NS) (fig 2; fig 6).^78^

#### Very low quality evidence

The highest versus lowest sugar sweetened beverage consumption might increase the risk of asthma in children by 26% (odds ratio 1.26, 1.07 to 1.48).^79^ In a single article,^80^ both sugar sweetened beverage consumption (odds ratio 1.80, 1.23 to 2.63) and total sugar consumption (1.22, 1.04 to 1.42) were associated with an increased risk of attention deficit/hyperactivity disorder. In addition, the results from a meta-analysis of 10 observational studies showed a significant inverse association between sugar sweetened beverage consumption and bone mineral density in adults (standardised mean difference −0.66, −1.01 to −0.31).^81^ Subgroup analysis according to sex showed a significant harmful effect of sugar sweetened beverage consumption on bone mineral density in females (standardised mean difference −0.50, −0.87 to −0.13) but no association in males.^81^ For dental diseases, a single article found a harmful association between sugar sweetened beverage consumption and the incidence of dental caries (odds ratio 1.72, 1.41 to 2.09) and dental erosion (1.77, 1.28 to 2.43) when comparing never/low with moderate/high consumption.^17^ Additionally, sugar sweetened beverage consumption was positively associated with the risk of non-alcoholic fatty liver disease (risk ratio 1.39, 1.29 to 1.50).^16^ Fructose intake was associated with increased intrahepatocellular lipids (standardised mean difference 0.45, 0.18 to 0.72) (fig 2; fig 6).^78^

Sugar sweetened beverage consumption was not associated with the risk of chronic kidney disease.^82^ In addition, maternal increased free sugar intake during pregnancy was not associated with the risk of asthma in offspring (supplementary table D).^83^

### Heterogeneity

We reanalysed the heterogeneity in 69% of all health outcomes by a random or fixed effects model. Reanalysis found that approximately 46% of the health outcomes that we reanalysed had significant heterogeneity (I^2^>50% or P value of Cochran’s Q test <0.1). The heterogeneity of most outcomes could be explained by some potential factors, including setting, region, ethnicity, sex, age, study quality, study design, sample size, duration of follow-up, and adjustment for confounding factors. Of the 26 outcomes that we could not reanalyse, approximately 54% had significant heterogeneity and 4% did not report the results of the heterogeneity evaluation.

### Assessment of risk of bias

We conducted Egger’s test for 23% of the outcomes in our reanalysis, which found evidence of publication bias in three outcomes—type 2 diabetes mellitus (sugar sweetened beverages) (P=0.016), overall cancer risk (P=0.005), and hypertension in adults (sugar sweetened beverages) (P=0.02). For outcomes that we could not reanalyse, publication bias was detected for cardiovascular disease mortality (sugar sweetened beverages), non-alcoholic fatty liver disease, obesity in adults, and change in body weight (one year) by statistical test or funnel plot. The remaining outcomes did not show significant publication bias or did not report an evaluation for publication bias.

### AMSTAR, GRADE, and evidence classification

The median AMSTAR score of all health outcomes was 8 (range 3-11; interquartile range 8-9.25) (supplementary table E). Supplementary table F provides the detailed AMSTAR scores for each outcome. All evidence from meta-analyses of cohorts, population based case-control studies, and cross sectional studies is graded as “low” or “very low” quality owing to the observational study design and factors for quality downgrade (significant risk of bias, inconsistency, indirectness, imprecision, and potential publication bias). Among the nine meta-analyses of randomised controlled trials, four (liver fat accumulation, muscle fat accumulation, serum uric acid (fructose), and change in body weight (sugar sweetened beverages)) were downgraded as “moderate” quality given the imprecision, and the remaining (alanine transaminase, intrahepatocellular lipids, postprandial triglycerides, change in body mass index in adults, and change in body weight (fructose)) were downgraded as “low” or “very low” owing to the risk of bias, inconsistency, indirectness, or imprecision (supplementary table E). Supplementary Table G shows the detailed GRADE classification for each outcome. In terms of evidence classification, type 2 diabetes mellitus (sugar sweetened beverages), hyperuricaemia (fructose), obesity in children (sugar sweetened beverages), coronary heart disease, hypertension in adults (sugar sweetened beverages), dental caries, depression, and non-alcoholic fatty liver disease were graded as class II. For the remaining 75 outcomes, 15 (18.1%) were graded as class III, 26 (31.3%) were graded as class IV, and 34 (41.0%) were identified as non-significant (supplementary table E). Sensitivity analyses of each outcome graded as class II did not alter the direction or significance of the association.

## Discussion

### Principal findings and possible explanations

Dietary sugar consumption is harmfully associated with multiple health outcomes across various measurements of exposure, including high versus low, never/low versus moderate/high, any versus none, or an extra increment of sugars per day (or week) versus none. We identified 73 meta-analyses and 83 health outcomes from 8601 unique articles, including 74 unique outcomes in meta-analyses of observational studies and nine unique outcomes in meta-analyses of randomised controlled trials.

Dietary sugar consumption had harmful associations with endocrine and metabolic outcomes, including changes in body mass index in children,^53^ changes in body weight,^53^ changes in body weight (one year),^53^ gout,^11^ ^13^ ^58^ high density lipoprotein cholesterol,^60^ hyperuricaemia,^11^ ^59^ latent autoimmune diabetes in adults,^30^ low density lipoprotein cholesterol,^60^ metabolic syndrome,^61^ obesity in children,^3^ obesity in adults,^1^ serum uric acid,^57^ type 2 diabetes mellitus,^6^ liver fat accumulation,^54^ and muscle fat accumulation.^54^ In addition, harmful associations between dietary sugar consumption and cardiovascular outcomes were also observed, including coronary heart disease,^10^ cardiovascular disease,^8^ cardiovascular disease mortality,^68^ ^71^ hypertension in children and adolescents,^69^ hypertension in adults,^70^ myocardial infarction,^66^ change in systolic blood pressure in children and adolescents,^69^ and stroke.^10^ Significant harmful associations between dietary sugar consumption and a higher risk of cancer were observed for breast cancer,^19^ breast cancer mortality,^18^ hepatocellular carcinoma,^18^ prostate cancer,^18^ pancreatic cancer,^72^ overall cancer risk,^18^ and overall cancer mortality.^18^ Finally, harmful associations existed between dietary sugar consumption and all cause mortality,^76^ asthma in children,^79^ attention deficit/hyperactivity disorder,^80^ bone mineral density,^81^ dental caries,^17^ dental erosion,^17^ depression,^77^ non-alcoholic fatty liver disease,^16^ and intrahepatocellular lipids.^78^

In general, no reliable evidence shows beneficial associations between dietary sugar consumption and any health outcomes, apart from glioma (sugar sweetened beverages),^18^ total cholesterol (sugar sweetened beverages),^60^ type 2 diabetes mellitus (sucrose),^62^ and cardiovascular disease mortality (sucrose).^71^ However, these favourable associations are not supported by strong evidence, and the interpretation of these results should be done with caution. For the decreased risk of glioma, evidence for this came from only two cohort studies, and no studies have shown that sugar sweetened beverage consumption is a protective factor to lower the incidence of cancer. High sugar sweetened beverage consumption was associated with lower total cholesterol concentrations. However, subgroup analysis indicated that sugar sweetened beverage consumption was associated with higher total cholesterol concentrations in studies with sugar sweetened beverage consumption >750 g/day and studies involving adolescents. Therefore, potential confounders, including region, sugar sweetened beverage dose, sample size, and sex, should be considered in explaining the association between sugar sweetened beverage consumption and total cholesterol concentrations. In terms of the protective effect of sucrose intake on type 2 diabetes mellitus and cardiovascular disease mortality, we note that sucrose tends to be found more in solid foods than in sugar sweetened beverages, including grains and grain based products, fruit and fruit products, and sweetened dairy and dairy products.^84^ ^85^ ^86^ These main sources of sucrose have shown beneficial associations (for example, whole grain cereals, fruit, and yogurt) with type 2 diabetes mellitus and cardiovascular disease mortality.^87^ ^88^ ^89^ ^90^ ^91^ ^92^ Therefore, the protective association between sucrose intake and type 2 diabetes mellitus and cardiovascular disease mortality may reflect important contributions from these other food sources rather than sucrose.^62^ ^71^ Further large scale, prospective studies are warranted to evaluate the association of sucrose intake with type 2 diabetes mellitus and cardiovascular disease mortality and to clarify the possible underlying mechanisms.

Our umbrella review showed harmful associations between dietary sugar consumption and a range of cardiometabolic diseases, especially weight gain, ectopic fat accumulation, obesity, and cardiovascular disease, which can largely be attributed to excessive consumption of fructose containing sugars. In response to the intake of large carbohydrates, fructose could enhance hepatic lipogenic capacity by inducing hepatic master transcription factors.^93^ ^94^ ^95^ Moreover, an animal study found that dietary fructose could be converted to microbial acetate by the gut microbiota, which may enhance hepatic lipogenesis by supplying lipogenic acetyl-CoA independently of ATP citrate lyase.^96^ Intermediate products such as diacylglycerols generated during the process of lipogenesis may impair insulin signalling in the liver and peripheral tissues and then lead to insulin resistance.^97^ Subsequently, it may promote ectopic fat deposition in the liver and muscle.^98^ ^99^ Dietary fructose may also inhibit fatty acid oxidation in the liver by impairing mitochondrial size and function and acetylation of the rate limiting enzyme.^100^ A recent animal study showed that dietary fructose improves the survival of intestinal cells and increases the length of intestinal villus in mouse models, resulting in an expanded surface area of the gut and increased nutrient absorption and adiposity in mice.^101^ Furthermore, fructose contained in sugar sweetened beverages is suggested to likely induce the onset of obesity by reducing resting energy expenditure and promoting leptin resistance.^102^ ^103^ In addition, sugar sweetened beverages are associated with less satiety compared with solid food containing the same amount of calories, which may stimulate appetite and induce excessive calorie consumption, liver fat accumulation, and insulin resistance in the long term.^104^ This hypothesis is confirmed by several clinical trials conducted in healthy adults, which found that sugar sweetened beverage consumption results in more caloric intake and weight gain than artificially sweetened beverages.^105^ ^106^ ^107^ Additionally, a recent double blind, randomised controlled trial carried out in 94 healthy men suggested that consumption of sugar sweetened beverages containing fructose might induce a significant change in the low density lipoprotein particle distribution towards smaller, more atherogenic particles, partially mediating the associations of sugar sweetened beverage consumption with dyslipidaemia and cardiovascular disease.^108^

Another important mechanism to explain the associations between dietary sugar consumption and cardiometabolic diseases involves uric acid synthesis. Many studies have confirmed that excessive fructose consumption can promote uric acid synthesis by inducing degradation ATP to AMP, a substrate for uric acid production.^109^ ^110^ ^111^ Fructose phosphorylation in the liver uses ATP to convert fructose into fructose-1-phosphate and leads to phosphate depletion, which limits the regeneration of ATP from ADP. Then, ADP is converted to AMP and consequently induces the synthesis of uric acid.^57^ In addition, fructose induced hyperinsulinaemia and insulin resistance may also result in higher serum uric acid by reducing the excretion of uric acid.^110^ ^112^ ^113^ Hyperuricaemia is a precursor to gout.^109^ ^110^ The positive associations between gout, hyperuricaemia, and other cardiometabolic diseases, such as hypertension, type 2 diabetes mellitus, and cardiovascular disease, have been proposed for a long time.^114^ ^115^ Hyperuricaemia has been shown to precede the occurrence of type 2 diabetes mellitus and obesity.^27^ Mechanistically, hyperuricaemia could induce renal microvascular alteration, chronic sodium retention, reduction in nitric oxide concentrations in endothelial cells, and the activation of the renin-angiotensin system, which may account for the association between fructose containing sugar consumption and cardiovascular disease.^114^ ^116^ ^117^ ^118^

Until now, the evidence for the association between dietary sugar consumption and the risk of cancer has remained limited and controversial.^27^ In 2018 the World Cancer Research Fund/American Institute for Cancer Research (WCRF/AICR) reported that evidence was limited for the associations between consumption of sugars and food containing sugars and the risk of colorectal cancer.^119^ However, at the same time, this report recommended reducing or avoiding sugar sweetened beverage consumption for the prevention of breast cancer.^119^ Evidence from this umbrella review supports the recommendations from the WCRF/AICR to some extent. In our study, although eight of the 25 cancer outcomes were identified as being positively associated with dietary sugar consumption (seven exposure factors were sugar sweetened beverages, and one was fructose), only evidence of hepatocellular carcinoma (sugar sweetened beverages) and pancreatic cancer (fructose) were rated as “low” quality because of the magnitude of effect or dose-response gradient, and the remaining outcomes were all rated as “very low” quality. As a result, caution is warranted when explaining the significant associations between dietary sugar consumption and some cancer risks.

The effect of dietary sugars on obesity might partly explain their association with the risk of cancer.^21^ As mentioned previously, dietary sugar consumption, especially sugar sweetened beverage consumption, is convincingly associated with the risk of obesity weight gain,^1^ ^3^ ^53^ which in turn is regarded as a strong risk factor for various cancers.^21^ ^119^ Another pathway mediating the association between dietary sugar consumption and the risk of cancer might involve a high glycaemic index or glycaemic load. The glycaemic index has been associated with the risk of type 2 diabetes mellitus,^120^ which may be involved in carcinogenesis of the breast, prostate, liver, bladder, and endometrium.^120^ ^121^ Moreover, excessive fructose consumption might lead to intestinal flora disturbance and intestinal barrier deterioration, which promote the development of metabolic endotoxaemia, inflammation, and lipid accumulation, finally leading to colorectal carcinogenesis.^20^ ^122^ ^123^ A recent animal study showed that high fructose corn syrup intake could induce intestinal tumourigenesis in mice by expediting glycolysis and de novo lipogenesis. The mice treated with the syrup had a substantially increased tumour size and tumour grade, independent of obesity and metabolic syndrome.^124^ Considering the different mechanisms of site specific cancers, further prospective studies that explore the definite associations between sugar consumption and cancer risk for diverse cancer types and ethnic groups are warranted.^27^

On the other hand, dietary sugar consumption has also been shown to be negatively associated with some neuropsychiatric diseases, such as depression and attention deficit/hyperactivity disorder.^77^ ^80^ Several biological mechanisms might be involved in these associations. Data from an animal study showed that a high fructose diet might alter behaviour, hypothalamic-pituitary-adrenal axis function, and the hypothalamic transcriptome in male Wistar rats, inducing anxiety-like behaviour and depressive-like behaviour.^125^ Furthermore, sugar consumption has been suggested to stimulate the secretion of endogenous opioids in the nucleus accumbens and to stimulate the dopaminergic reward system.^27^ Evidence of sugar dependence in an animal model indicated that similarly to addiction to morphine and cocaine, rats with intermittent sugar intake had decreased concentrations of dopamine D2 receptor mRNA in the nucleus accumbens and showed the characteristics of addictive-like behaviours called sugar addiction.^27^ ^126^

In addition, the adverse association between sugar consumption and bone mineral density might be attributed to the increased loss of urinary calcium and imbalance in calcium homoeostasis induced by high sugar intake.^127^ As well as the negative effect of sugars, phosphate, acidity, and caffeine contained in sugar sweetened beverages are three other major factors that affect bone metabolism.^81^ We note that for the link between sugar sweetened beverages and bone mineral density, stratification analysis by gender showed a significant harmful effect of sugar sweetened beverages on bone mineral density in females but not in males.^81^ These diverse findings indicated that sugar sweetened beverage consumption had a more detrimental effect on female bone health than on male bone health because women generally have smaller bones and lower bone strength and are therefore more susceptible to osteoporosis.^128^ Moreover, the high acidity of sugar sweetened beverages is also thought to be an important factor in promoting dental caries and tooth erosion.^129^ ^130^ ^131^

Of the subgroup analyses conducted in this umbrella review, the most noteworthy is the stratification according to region, as several health outcomes showed a regional discrepancy, including overall cancer mortality, high density lipoprotein cholesterol, low density lipoprotein cholesterol, and total cholesterol. Potential reasons for these discrepancies may include regional differences in sugar consumption and culture. According to the report conducted in 2010 for the quantification of global, regional, and national consumption of sugar sweetened beverages in 187 countries, consumption among Asian countries was lower than that among European and American countries.^33^ The consumption of sugar sweetened beverages was highest in the Caribbean and lowest in East Asia and Oceania.^33^ In addition, cultural factors have been shown to potentially cause different dietary quality and health inequalities by affecting food preferences or choices.^132^ Regional cultural diversity in lifestyle and sociodemographic factors also plays an important role in dietary sugar consumption, which may partly explain the different relations between sugar consumption and disease risk in ethnically diverse populations.^132^ ^133^ On the other hand, subgroup analyses with adjustment for confounding factors should also be considered. High consumption of sugars, especially sugar sweetened beverages, may be a marker of an unhealthy diet and lifestyle.^9^ ^66^ People who consumed sugar sweetened beverages more frequently were likely to ingest more total and saturated fat, carbohydrate, and sodium and less fruit, fibre, dairy products, and wholegrain foods.^134^ ^135^ ^136^ ^137^ ^138^ This dietary pattern was also associated with more frequent smoking and drinking, lower physical activity levels, and more time spent watching television.^137^ ^138^ Therefore, the role of these confounding factors should be taken into consideration when explaining the association between sugar consumption and burden of disease.

### Strengths and weaknesses of study and in relation to other studies

This umbrella review first reported a comprehensive summary of the current evidence from previous meta-analyses of observational studies and randomised controlled trials for the association between dietary sugar consumption and all health outcomes. Given the high levels of dietary sugar consumption worldwide, this study has clinical and social significance for developing preventive strategies against excessive sugar consumption, especially for children and adolescents. This study was carried out on the basis of systematic methods in which independent literature searching, study selection, and data extraction by two authors were involved. If the data were sufficient, we reanalysed the risk ratio, odds ratio, weighted mean difference, or standardised mean difference with 95% confidence intervals through random or fixed effects models and evaluated the heterogeneity and publication bias for each included meta-analysis. Furthermore, we used three standard approaches, AMSTAR, GRADE, and evidence classification criteria, to assess the methodological quality (AMSTAR), strength (GRADE) and classification (evidence classification criteria) of evidence for each health outcome and to evaluate our confidence in the estimates. Interestingly, in our umbrella review, the GRADE rating of several health outcomes was not completely consistent with the results of evidence classification. As we know, evidence classification criteria are a completely objective classification standard, whereas the GRADE rating has a certain degree of subjectivity.^139^ Therefore, both the GRADE rating and evidence classification criteria should be considered when evaluating evidence and making recommendations.

Original studies included in meta-analyses used different methods of food intake investigation, including food records, 24 hour dietary recall, food frequency questionnaires, and dietary history. All of these are associated with an unavoidable measurement bias even if validated methods are used.^3^ This limitation is common to all major epidemiological studies carried out worldwide in this field.^21^ In addition, most studies focused on beverages pre-sweetened before purchase.^9^ For instance, in the Nurses’ Health Study, coffee with sugars was excluded from sugar sweetened beverages, which might affect the reliability of the association.^137^ Similarly, another limitation of our study was that we could not evaluate sugar intake in some foods that potentially contain sugars, such as chocolate and ice cream, because of a failure to extract data on sugar consumption. Furthermore, the types of sugar sweetened beverages and dosage of their consumption varied in the original studies. In this umbrella review, most meta-analyses produced summary effects from original studies that measured exposure to dietary sugars through the number of servings a day. However, in some original studies, the number of millilitres a day, grams a day, times a day, times a week, times a month, servings a week, or servings a month were used to estimate sugar consumption, which may partly explain the origin of heterogeneity in meta-analyses. Therefore, dose-response analysis and stratification analysis by sugar sweetened beverage types were unavailable for most outcomes owing to diverse measurements of sugar sweetened beverage consumption in the original studies. Consumption of sugars in sugar sweetened beverages is generally accompanied by the ingestion of some other chemical compounds, such as 4-methylimidazole,^140^ ^141^ pesticides,^142^ ^143^ artificial sweeteners,^144^ sodium benzoate,^79^ and sulfites,^79^ which may confuse the effect of sugars and therefore should be regarded as potential confounding factors.

We reviewed details of competing interest and funding disclosures from meta-analyses included in this umbrella review. Only two meta-analyses were funded by companies that produce sugar sweetened beverages.^65^ ^145^ Among them, the meta-analysis conducted by Wang and colleagues was selected for data extraction and is shown in summary tables.^65^ Therefore, caution is warranted when explaining the non-significant association between fructose intake and postprandial triglycerides. Another meta-analysis was not selected for data extraction,^145^ and the list of all meta-analyses not selected for data extraction and reanalysis are available if needed. We did not investigate the original studies included in each meta-analysis and therefore could not confirm whether these studies had a competing interest with companies associated with the sugar industry.^42^

The harmful association between dietary sugar consumption and multiple health outcomes observed in our umbrella review is supported by several large scale prospective cohort studies published in recent years. The first was a large prospective cohort study conducted using the results of the French NutriNet-Santé cohort (2009-17), which included 101 257 participants with an average age of 42.2.^21^ During the eight year follow-up period, a total of 2193 cases of cancer were reported, including 693 cases of breast cancer. A harmful association was found between sugar sweetened beverage consumption and the risk of overall cancer (hazard ratio 1.18, 1.10 to 1.27) and breast cancer (1.22, 1.07 to 1.39). No significant association was observed for sugar sweetened beverage consumption and the risk of prostate, colorectal, and lung cancer.^21^ In this umbrella review, however, the highest versus lowest sugar sweetened beverage consumption was associated with a 17% increased risk of prostate cancer, without a dose-response gradient. Notably, the non-significant association between sugar sweetened beverage consumption and the risk of colorectal cancer observed both in this study and in our umbrella review was inconsistent with another cohort conducted in women.^20^ In the Nurses’ Health Study II (1991-2015), the authors prospectively explored the association of sugar sweetened beverage consumption in adulthood and adolescence with the risk of early onset colorectal cancer among 95 464 women. A total of 109 cases of early onset colorectal cancer were confirmed during follow-up. Compared with women who consumed less than one serving a week of sugar sweetened beverages in adulthood, those who consumed two or more servings a day had a 118% higher risk of early onset colorectal cancer (risk ratio 2.18, 1.10 to 4.35). Each one serving a day increment of sugar sweetened beverage consumption was associated with a 16% (risk ratio 1.16, 1.00 to 1.36) increased risk of early onset colorectal cancer.^20^ In addition, another prospective cohort study showed that excessive consumption of sugars and sugar sweetened beverage during adolescence was significantly associated with the risk of colorectal adenoma (odds ratio 1.20, 1.04 to 1.39).^146^ Each one serving a day increase in sugar sweetened beverage consumption was associated with 11% (odds ratio 1.11, 1.02 to 1.20) and 30% (1.30, 1.08 to 1.55) higher risks of total colorectal adenoma and rectal adenoma, respectively.^146^ Given that the association between sugar consumption and colorectal cancer risk remains controversial, further well designed, large scale prospective studies are needed to clarify it.

The positive associations between sugar sweetened beverage consumption and the risk of mortality detected in this umbrella review were supported by a prospective cohort study of 118 363 people followed for 34 years in the US, during which time 36 436 deaths were documented.^147^ After adjustment for diet and lifestyle confounders, the consumption of two or more servings of sugar sweetened beverages a day was linked with a 21% (hazard ratio 1.21, 1.13 to 1.28) higher risk of total mortality, a 31% (1.31, 1.15 to 1.50) higher risk of cardiovascular disease mortality, and a 16% (1.16, 1.04 to 1.29) higher risk of cancer mortality.^147^ On the other hand, a prospective cohort study of 120 343 UK participants followed for 8.4 years confirmed the harmful association of added sugar consumption with the risk of type 2 diabetes mellitus.^148^ A dietary pattern high in added sugars was associated with a higher incidence of type 2 diabetes mellitus (hazard ratio 1.09, 1.06 to 1.12) after adjustment for confounders.^148^ Similar to their findings, we observed a strongly significant association between consumption of sugar sweetened beverages (one of the main sources of added sugars) and the risk of type 2 diabetes mellitus.

### Conclusions and recommendations

This umbrella review shows that high dietary sugar consumption, especially intake of sugars that contain fructose, is harmfully associated with large numbers of health outcomes. Evidence for the harmful associations between dietary sugar consumption and changes in body weight (sugar sweetened beverages), ectopic fat accumulation (added sugars), obesity in children (sugar sweetened beverages), coronary heart disease (sugar sweetened beverages), and depression (sugar sweetened beverages) seems to be more reliable than that for other outcomes. Evidence of the association between dietary sugar consumption and cancer remains limited but warrants further research. In combination with the WHO and WCRF/AICR recommendations and our findings, we recommend reducing the consumption of free sugars or added sugars to below 25 g/day (approximately six teaspoons a day) and limiting the consumption of sugar sweetened beverages to less than one serving a week (approximately 200-355 mL/week).^38^ ^119^ To change sugar consumption patterns, especially for children and adolescents, a combination of widespread public health education and policies worldwide is urgently needed.

## What is already known on this topic

Sugar consumption could have negative effects on health, especially obesity, diabetes, cardiovascular disease, hyperuricaemia, gout, ectopic fatty accumulation, dental caries, and some cancers
Deficiencies in study design, varying measurements, inconsistent findings, and different definitions of exposure make drawing final conclusions on associations difficult
Comprehensive evaluation of the quality of existing evidence on the associations of sugar consumption with all health outcomes is needed

## What this study adds

High dietary sugar consumption is generally more harmful than beneficial for health, especially in cardiometabolic disease
Evidence of the association between dietary sugar consumption and cancer remains limited but warrants further research
Existing evidence is mostly observational and of low quality, and further randomised controlled trials are needed

## Data Availability

The list of all meta-analyses not selected for data extraction and reanalysis is available if needed.

## Web extra

Extra material supplied by authors

Web appendix: Supplementary materials

## Notes

Contributors: YH, ZYC, BC, and, JZL are joint first authors and contributed equally to this work. QW, DHC, and LRL are joint corresponding authors and contributed equally to this work. YH, ZYC, BC, and JZL conducted study selection, data extraction, and analysis and wrote the manuscript. QW, DHC, and LRL designed the study, supervised the project, and revised the manuscript. XY, JL, WW, TTD, HYC, YW, RYW, PZW, JBG, QD, and CFL assisted with detailed statistical analysis. All authors reviewed and approved the final version of the manuscript. LRL is the guarantor. The corresponding authors (QW, DHC, and LRL) attest that all listed authors meet authorship criteria and that no others meeting the criteria have been omitted.

The lead author affirms that the manuscript is an honest, accurate, and transparent account of the study being reported; that no important aspects of the study have been omitted; and that any discrepancies from the study as planned (and, if relevant, registered) have been explained.

Dissemination to participants and related patients and public communities: After publication, the findings of this review will be disseminated to appropriate audiences such as academia, clinicians, policy makers, and the general public, through various channels including blogs, press releases, and social media.

Provenance and peer review: Not commissioned; externally peer reviewed.
