# Supplementary material for: Dietary sugar consumption and health: umbrella review
Source: BMJ. 2023 Apr 5;381:e071609. doi: 10.1136/bmj-2022-071609 (PMC10074550; doi:10.1136/bmj-2022-071609)
Supplement: Supplementary file 1 — Web appendix: Supplementary materials [file huay071609.ww1.pdf]

Supplementary Table A. Associations between dietary sugars consumption and endocrine & metabolic outcomes.

Outcome	Category	Total eligible MA	Included MA	No. of cases/total	MA metric	Estimates [95% CI]	No. of studies (T/R/C/P)	Effects model	I^2; *Q* test *P* value	Egger test *P* value
Significant associations										
Δ BMI[a]	SSB	3	Malik 2013	NA/25,745	WMD[b]	0.07 [0.01-0.12]	20/0/20/0	random	91.6%; < 0.001	NA
Δ Body weight	SSB	2	Malik 2013	NA/292[c]	WMD[d]	0.85 [0.50-1.20]	6/6/0/0	fixed	0.0%; 0.780	NA
Δ Body weight (one year)	SSB	1	Malik 2013	NA/174,252	WMD[b]	0.22 [0.09-0.34]	8/0/8/0	random	70.2%; 0.001	NA
Gout	SSB	3	Ebrahimpour-koujan 2020	NA/141,091	RR[d]	1.35 [1.18-1.55]	3/0/3/0	random	40.1%; 0.188	0.766
Gout (dose-response)	SSB	2	Ayoub-Charette 2019	1,533/125,299	RR[e]	1.04 [1.02-1.07]	2/0/2/0	random	0%; 0.52	NA
HDL-C	SSB	1	Nikniaz 2021	8,548/24,119[f]	WMD[d]	-1.46 [-2.25-(-0.67)]	14/0/1/13	random	99.3%; < 0.001	0.65
Hyperuricemia	SSB	1	Ebrahimpour-koujan 2020	NA/63,246[c]	RR[d]	1.35 [1.19-1.52]	6/0/0/6	random	41.4%; 0.129	0.214
LADA	SSB	1	El-Malky 2020	1,862/8,133[c]	OR[d]	1.26 [1.12-1.41]	6/0/0/6	random	73.1%; < 0.001	0.75
LDL-C	SSB	1	Nikniaz 2021	7,185/18,668[f]	WMD[d]	1.21 [0.23-2.20]	9/0/1/8	random	97.2%; < 0.001	0.65
Metabolic syndrome	SSB	4	Semnani-Azad 2020	7,138/19,079	RR[g]	1.14 [1.05-1.23]	6/0/6/0	random	79.3%; 0.001	NA
Obesity[a]	SSB	1	Morenga 2013	NA/12,317	OR[h]	1.55 [1.32-1.82]	7/0/7/0	random	0%; 0.69	NA
Obesity	SSB	2	Qin 2020	11,821/56,579	RR[i]	1.12 [1.05-1.19]	7/0/7/0	random	67.7%; 0.005	0.007
Serum uric acid	SSB	1	Ebrahimpour-koujan 2021	NA/26,260[c]	WMD[d]	0.18 [0.11-0.25]	6/0/0/6	random	0.0%; 0.698	NA
T2DM	SSB	9	Meng 2021	15,768/445,040	RR[b]	1.27 [1.15-1.41]	16/0/16/0	random	80.8%; < 0.001	0.016
TC	SSB	1	Nikniaz 2021	6,845/17,728[c]	WMD[d]	-2.49 [-2.89-(-2.10)]	7/0/0/7	random	77.3%; < 0.001	0.51
Gout	Fructose	3	Jamnik 2016	1,553/125,299	RR[d]	1.62 [1.28-2.03]	2/0/2/0	fixed	0%; 0.33	NA
Hyperuricemia	Fructose	3	Li 2018	2,655/17,684[c]	OR[d]	1.85 [1.66-2.07]	4/0/0/4	fixed	31%; 0.23	NA
T2DM	Sucrose	1	Tsilas 2017	3,833/107,972	RR[j]	0.92 [0.85-0.99]	6/0/6/0	random	12%; 0.34	NA
Liver fat accumulation	Added sugars	1	Ma 2016	NA/104[c]	SMD[h]	0.93 [0.64-1.21]	8/8/0/0	random	0.0%; 0.754	> 0.05
Muscle fat accumulation	Added sugars	1	Ma 2016	NA/80[c]	SMD[h]	0.63 [0.23-1.04]	5/5/0/0	random	41.7%; 0.143	> 0.05
Non-significant associations										
Δ BMI	SSB	1	Mattes 2011	NA/2,722[c]	SMD[k]	-0.004 [-0.079-0.072]	6/6/0/0	fixed	22.36%; 0.266	NA
TG	SSB	1	Nikniaz 2021	8,232/21,392[f]	WMD[d]	5.29 [-0.52-11.10]	13/0/1/12	random	99.7%; < 0.001	0.97
Large waist circumference	SSB	1	Ardeshirlarijani 2021	6,580/24,007	RR[d]	1.14 [0.86-1.51]	10/0/10/0	random	90.8%; < 0.001	NA
Δ Body weight	Fructose	1	Sievenpiper 2012	201/216[c]	WMD[h]	-0.13 [-0.37-0.10]	13/13/0/0	random	8%; 0.37	> 0.05
Postprandial TG	Fructose	1	Wang 2014	NA/154[c]	SMD[h]	0.30 [0.00-0.60]	5/5/0/0	random	70%; 0.01	0.514
Serum uric acid	Fructose	1	Wang 2012	169/172[c]	WMD[h]	1.28 [-6.65-9.22]	9/9/0/0	random	1%; 0.43	0.95
T2DM	Fructose	1	Tsilas 2017	3,833/107,972	RR[d]	1.04 [0.84-1.29]	6/0/6/0	random	71%; < 0.01	NA
T2DM	Total sugars	1	Tsilas 2017	13,727/105,846	RR[d]	0.91 [0.76-1.09]	12/0/12/0	random	76%; < 0.001	0.4

MA, meta-analysis; CI, confidence interval; T, total No. of studies; R, randomized controlled trial; C, cohort studies; P, population-based case-control and/or cross-sectional studies; Δ, final value − baseline value; BMI, body mass index; SSB, sugar-sweetened beverage; WMD, weighted mean difference; NA, not available; RR, relative risk; HDL-C, high-density lipoprotein cholesterol; LADA, latent autoimmune diabetes in adults; OR, odds ratio; LDL-C, low-density lipoprotein cholesterol; T2DM, type 2 diabetes mellitus; TC, total cholesterol; SMD, standardized mean difference; TG, triglycerides.

[a] Children.

[b] 1 serving/d increment.

[c] Case/control.

[d] Highest versus lowest.

[e] 1 serving/week increment.

[f] Case-control/cross-sectional and cohort studies.

[g] 355 mL/d increment.
[h] Any versus none.
[i] 250 mL/d increment.
[j] 25 g/d increment.
[k] Reducing SSB consumption versus not reducing.

Supplementary Table B. Associations between dietary sugars consumption and cardiovascular outcomes.

Outcome	Category	Total eligible MA	Included MA	No. of cases/total	MA metric	Estimates [95% CI]	No. of studies (T/C/P)	Effects model	I^2; *Q* test *P* value	Egger test *P* value
Significant associations										
CHD	SSB	2	Bechthold 2017	9,135/173,752	RR[a]	1.17 [1.11-1.23]	4/4/0	random	0%; 0.66	NA
CVD	SSB	4	Yin 2021	16,999/329,791	RR[b]	1.08 [1.02-1.14]	7/7/0	random	43.0%; 0.07	NA
CVD mortality	SSB	4	Zhang 2021	24,365/898,005	HR[b]	1.08 [1.04-1.12]	13/13/0	random	16.4%; 0.28	0.066
Hypertension[c]	SSB	1	Farhangi 2020	19,375/70,728[d]	OR[e]	1.36 [1.14-1.63]	5/0/5	random	0.0%; 0.976	0.127
Hypertension	SSB	7	Liu 2019	120,553/427,630	RR[f]	1.11 [1.09-1.13]	13/13/0	random	73%; < 0.001	0.02
MI	SSB	1	Narain 2016	NA/171,189	RR[e]	1.19 [1.09-1.31]	4/4/0	random	0%; 0.56	NA
Δ SBP[c]	SSB	1	Farhangi 2020	25,772/84,054[g]	WMD[e]	1.67 [1.02-2.32]	15/4/11	random	99.8%; < 0.001	0.267
Stroke	SSB	2	Bechthold 2017	12,160/289,406	RR[a]	1.07 [1.02-1.12]	6/6/0	random	0%; 0.59	NA
CVD mortality	Fructose	1	Khan 2019	10,894/353,751	RR[e]	1.08 [1.01-1.15]	2/2/0	random	0%; 0.90	NA
CVD mortality	Sucrose	1	Khan 2019	10,894/353,751	RR[e]	0.94 [0.89-0.99]	2/2/0	random	0%; 0.76	NA
CVD mortality	Total sugars	1	Khan 2019	11,393/362,607	RR[e]	1.09 [1.02-1.17]	4/4/0	random	0%; 0.97	NA
Non-significant associations										
Δ DBP[c]	SSB	1	Farhangi 2020	25,702/83,741[g]	WMD[e]	0.31 [-0.13-0.76]	14/4/10	random	99.7%; < 0.001	0.592
Heart failure	SSB	1	Bechthold 2017	5,493/46,890	RR[e]	1.11 [0.88-1.39]	2/2/0	random	81%; 0.02	NA
Hypertension	Fructose	1	Jayalath 2014	58,162/97,315	RR[e]	1.02 [0.99-1.04]	3/3/0	random	0%; 0.59	0.632
CVD	Sucrose	1	Khan 2019	3,682/101,966	RR[e]	1.10 [0.99-1.22]	3/3/0	random	0%; 0.43	NA
CVD mortality	Added sugars	1	Khan 2019	11,725/365,484	RR[e]	1.03 [0.85-1.26]	4/4/0	random	75.1%; 0.007	NA
CVD	Total sugars	1	Khan 2019	8,174/167,430	RR[e]	1.01 [0.90-1.13]	8/8/0	random	0%; 0.86	NA

MA, meta-analysis; CI, confidence interval; T, total No. of studies; C, cohort studies; P, population-based case-control and/or cross-sectional studies; CHD, coronary heart disease; SSB, sugar-sweetened beverage; RR, relative risk; NA, not available; CVD, cardiovascular disease; HR, hazard ratio; OR, odds ratio; MI, myocardial infarction; Δ, final value − baseline value; SBP, systolic blood pressure; WMD, weighted mean difference; DBP, diastolic blood pressure.

[a] 250 mL/d increment.

[b] 1 serving/d increment.

[c] Children and adolescence.

[d] Case/control.

[e] Highest versus lowest.

[f] 355 mL/d increment.

[g] Case-control/cross-sectional and cohort studies.

Supplementary Table C. Associations between dietary sugars consumption and cancer outcomes.

Outcome	Category	Total eligible MA	Included MA	No. of cases/total	MA metric	Estimates [95% CI]	No. of studies (T/C/P)	Effects model	I^2; Q test P value	Egger test P value
Significant associations										
Breast cancer	SSB	2	Llaha 2021	4,464/169,424[a]	RR[b]	1.14 [1.01-1.30]	6/4/2	random	0.0%; 0.69	NA
Breast cancer mortality	SSB	1	Li 2021	NA	RR[b]	1.17 [1.03-1.34]	2/2/0	random	0%; 0.611	NA
Glioma	SSB	1	Li 2021	678/1,156,603	RR[b]	0.81 [0.66-0.99]	2/2/0	random	0%; 0.774	NA
Hepatocellular carcinoma	SSB	1	Li 2021	372/477,114[a]	RR[b]	2.00 [1.33-3.03]	2/1/1	random	0%; 0.526	NA
Prostate cancer	SSB	2	Li 2021	4,287/78,214	RR[b]	1.17 [1.07-1.28]	6/6/0	random	0%; 0.800	NA
Overall cancer risk	SSB	1	Li 2021	24,482/5,505,812	RR[c]	1.04 [1.01-1.09]	20/20/0	random	68.5%; 0.032	0.005
Overall cancer mortality	SSB	2	Li 2021	57,567/1,239,183	RR[b]	1.06 [1.00-1.12]	10/10/0	random	50.9%; 0.018	0.869
Pancreatic cancer	Fructose	1	Aune 2012	2,430/1,031,605	RR[d]	1.22 [1.08-1.37]	6/6/0	random	0%; 0.43	0.22
Non-significant associations										
Biliary track cancer	SSB	1	Li 2021	450/538,545	RR[b]	1.01 [0.90-1.13]	2/2/0	random	58.9%; 0.045	NA
Bladder cancer	SSB	1	Li 2021	3,039/236,548[a]	RR[b]	1.14 [0.98-1.33]	6/1/5	random	0%; 0.515	NA
Colon cancer	SSB	1	Zhang 2010	4,895/704,133	RR[e]	1.00 [0.91-1.10]	13/13/0	random	0%; 0.57	NA
Colorectal cancer	SSB	2	Li 2021	5,564/300,830	RR[b]	1.07 [0.97-1.18]	6/6/0	random	28.3%; 0.193	NA
Colorectal cancer mortality	SSB	1	Li 2021	5,564/300,830	RR[b]	1.09 [0.90-1.33]	6/6/0	random	64.7%; 0.006	NA
Endometrial cancer	SSB	1	Li 2021	762/58,632	RR[b]	1.21 [0.73-1.99]	2/2/0	random	63.6%; 0.064	NA
Esophageal cancer	SSB	1	Li 2021	2,705/486,641[a]	RR[b]	0.84 [0.63-1.12]	5/1/4	random	59%; 0.099	NA
Gastric cancer	SSB	1	Li 2021	620/517,156	RR[b]	0.94 [0.73-1.22]	2/2/0	random	0.0%; 0.637	NA
Hematologic malignancy	SSB	1	Li 2021	3,144/225,470	RR[b]	1.09 [0.92-1.30]	2/2/0	random	10.4%; 0.347	NA
Kidney cancer	SSB	1	Li 2021	1,624/810,545	RR[b]	1.14 [0.94-1.38]	2/2/0	random	0%; 0.511	NA
Lung cancer mortality	SSB	1	Li 2021	NA	RR[b]	0.99 [0.86-1.13]	2/2/0	random	0%; 0.557	NA
Nasopharyngeal carcinoma	SSB	1	Li 2021	683/482,157[a]	RR[b]	0.81 [0.66-1.00]	2/1/1	random	0%; 0.941	NA
Pancreatic cancer	SSB	4	Li 2021	4,830/1,829,463	RR[b]	1.16 [0.92-1.47]	6/6/0	random	71.7%; 0.003	NA
Prostate cancer mortality	SSB	1	Li 2021	NA	RR[b]	0.96 [0.79-1.17]	2/2/0	random	0%; 0.791	NA
Colorectal cancer	Added sugars	1	Galeone 2012	3,897/435,686	RR[b]	1.01 [0.90-1.13]	2/2/0	random	0.0%; 0.801	NA
Pancreatic cancer	Sucrose	1	Aune 2012	2,801/1,092,616	RR[d]	1.05 [0.92-1.19]	8/8/0	random	53%; 0.04	0.71
Ovarian cancer	Lactose	1	Liu 2015	NA	OR[b]	1.084 [0.924-1.272]	14/14/0	random	NA; NA	0.054

MA, meta-analysis; CI, confidence interval; T, total No. of studies; C, cohort studies; P, population-based case-control and/or cross-sectional studies; SSB, sugar-sweetened beverage; RR, relative risk; NA, not available; OR, odds ratio.

[a] Case-control/cross-sectional and cohort studies.

[b] Highest versus lowest.

[c] 1 serving/d increment.

[d] 25 g/d increment.

[e] 375 g/d increment.

Supplementary Table D. Associations between dietary sugars consumption and other outcomes.

Outcome	Category	Total eligible MA	Included MA	No. of cases/total	MA metric	Estimates [95% CI]	No. of studies (T/R/C/P)	Effects model	I^2; *Q* test *P* value	Egger test *P* value
Significant associations										
All-cause mortality	SSB	6	Pan 2021	NA/1,088,524	HR[a]	1.04 [1.02-1.06]	11/0/11/0	random	88%; < 0.001	NA
Asthma[b]	SSB	1	Al-Zalabani 2019	6,812/49,293[c]	OR[d]	1.26 [1.07-1.48]	7/0/1/6	random	65.9%; 0.012	NA
ADHD	SSB	1	Farsad-Naeimi 2020	NA/1,981[e]	OR[d]	1.80 [1.23-2.63]	2/0/0/2	fixed	51.1%; 0.153	NA
BMD	SSB	1	Ahn 2021	NA/4,312[e]	SMD[d]	-0.66 [-1.01-(-0.31)]	10/0/0/10	random	91%; < 0.01	NA
Dental caries	SSB	1	Valenzuela 2021	NA/17,892[c]	OR[f]	1.72 [1.41-2.09]	17/0/1/16	random	75.9%; < 0.001	NA
Dental erosion	SSB	1	Valenzuela 2021	NA/15,927[e]	OR[f]	1.77 [1.28-2.43]	13/0/0/13	random	90.9%; < 0.001	NA
Depression	SSB	2	Hu 2019	37,131/620,411[c]	RR[d]	1.31 [1.24-1.39]	10/0/4/6	random	29.2%; 0.176	0.726
NAFLD	SSB	3	Chen 2019	1,326/9,705[c]	RR[d]	1.39 [1.29-1.50]	12/0/1/11	fixed	42%; 0.06	NA
IHCL	Fructose	2	Chiu 2014	NA/60[e]	SMD[g]	0.45 [0.18-0.72]	5/5/0/0	random	55%; 0.07	> 0.05
ADHD	Total sugars	1	Farsad-Naeimi 2020	NA/25,945[c]	OR[d]	1.22 [1.04-1.42]	7/0/3/4	random	81.9%; < 0.001	NA
Non-significant associations										
CKD	SSB	2	Lo 2021	NA/25,455[c]	RR[d]	1.30 [0.88-1.94]	6/0/5/1	random	82.5%; < 0.001	0.259
ALT	Fructose	2	Chiu 2014	NA/164[e]	WMD[g]	0.15 [-1.51-1.82]	6/6/0/0	random	0%; 0.97	> 0.05
Asthma in offspring[h]	Free sugars	1	Gupta 2021	NA/20,048	OR[d]	1.07 [1.00-1.14]	2/0/2/0	random	0.0%; 0.387	NA

MA, meta-analysis; CI, confidence interval; T, total No. of studies; R, randomized controlled trial; C, cohort studies; P, population-based case-control and/or cross-sectional studies; SSB, sugar-sweetened beverage; HR, hazard ratio; NA, not available; OR, odds ratio; ADHD, Attention Deficit Hyperactivity Disorder; BMD, bone mineral density; SMD, standardized mean difference; RR, relative risk; NAFLD, non-alcoholic fatty liver disease; IHCL, intrahepatocellular lipids; CKD, chronic kidney disease; ALT, alanine aminotransferase; WMD, weighted mean difference.

[a] 250 mL/d increment.

[b] Children.

[c] Case-control/cross-sectional and cohort studies.

[d] Highest versus lowest.

[e] Case/control.

[f] Never/low versus moderate/high consumption.

[g] Any versus none.

[h] Maternal increased free sugar intake during pregnancy.

Supplementary Table E. Assessments of AMSTAR scores, GRADE classification and evidence classification.

Outcome	Category	Study	Study design	AMSTAR	GRADE	Evidence class
Endocrine & metabolic outcomes						
Δ Body weight	SSB	Malik 2013	RCT	8	moderate	IV
Liver fat accumulation	Added sugars	Ma 2016	RCT	8	moderate	IV
Muscle fat accumulation	Added sugars	Ma 2016	RCT	8	moderate	IV
Serum uric acid	Fructose	Wang 2012	RCT	9	moderate	NS
Obesity[a]	SSB	Morenga 2013	Cohort	9	low	II
Gout (dose-response)	SSB	Ayoub-Charette 2019	Cohort	9	low	III
Δ BMI[a]	SSB	Malik 2013	Cohort	8	low	IV
Δ Body weight	Fructose	Sievenpiper 2012	RCT	8	low	NS
Gout	SSB	Ebrahimpour-koujan 2020	Cohort	9	very low	III
Gout	Fructose	Jamnik 2016	Cohort	9	very low	III
HDL-C	SSB	Nikniaz 2021	Cohort, case-control and/or cross-sectional	10	very low	III
Hyperuricemia	SSB	Ebrahimpour-koujan 2020	Case-control and/or cross-sectional	9	very low	III
Hyperuricemia	Fructose	Li 2018	Case-control and/or cross-sectional	8	very low	II
LADA	SSB	El-Malky 2020	Case-control and/or cross-sectional	7	very low	III
Large waist circumference	SSB	Ardeshirlarijani 2021	Cohort	9	very low	NS
LDL-C	SSB	Nikniaz 2021	Cohort, case-control and/or cross-sectional	10	very low	IV
Metabolic syndrome	SSB	Semnani-Azad 2020	Cohort	10	very low	IV

Obesity	SSB	Qin 2020	Cohort	8	very low	III
Postprandial TG	Fructose	Wang 2014	RCT	9	very low	NS
Serum uric acid	SSB	Ebrahimpour-koujan 2021	Case-control and/or cross-sectional	10	very low	IV
T2DM	SSB	Meng 2021	Cohort	8	very low	II
T2DM	Sucrose	Tsilas 2017	Cohort	10	very low	IV
T2DM	Fructose	Tsilas 2017	Cohort	10	very low	NS
T2DM	Total sugars	Tsilas 2017	Cohort	10	very low	NS
TC	SSB	Nikniaz 2021	Case-control and/or cross-sectional	10	very low	IV
TG	SSB	Nikniaz 2021	Cohort, case-control and/or cross-sectional	10	very low	NS
Δ BMI	SSB	Mattes 2011	RCT	8	very low	NS
Δ Body weight (one year)	SSB	Malik 2013	Cohort	8	very low	IV
Cardiovascular outcomes						
CHD	SSB	Bechthold 2017	Cohort	9	low	II
MI	SSB	Narain 2016	Cohort	8	low	III
Hypertension	Fructose	Jayalath 2014	Cohort	9	low	NS
CVD	SSB	Yin 2021	Cohort	9	very low	IV
CVD	Sucrose	Khan 2019	Cohort	10	very low	NS
CVD	Total sugars	Khan 2019	Cohort	10	very low	NS
CVD mortality	SSB	Zhang 2021	Cohort	10	very low	III
CVD mortality	Fructose	Khan 2019	Cohort	10	very low	IV
CVD mortality	Sucrose	Khan 2019	Cohort	10	very low	IV
CVD mortality	Total sugars	Khan 2019	Cohort	10	very low	IV
CVD mortality	Added sugars	Khan 2019	Cohort	10	very low	NS

Heart failure	SSB	Bechthold 2017	Cohort	9	very low	NS
Hypertension	SSB	Liu 2019	Cohort	11	very low	II
Hypertension[b]	SSB	Farhangi 2020	Case-control and/or cross-sectional	8	very low	III
Stroke	SSB	Bechthold 2017	Cohort	9	very low	IV
Δ DBP[b]	SSB	Farhangi 2020	Cohort, case-control and/or cross-sectional	8	very low	NS
Δ SBP[b]	SSB	Farhangi 2020	Cohort, case-control and/or cross-sectional	8	very low	III
Cancer outcomes						
Pancreatic cancer	Fructose	Aune 2012	Cohort	6	low	III
Hepatocellular carcinoma	SSB	Li 2021	Cohort, case-control and/or cross-sectional	8	low	IV
Biliary track cancer	SSB	Li 2021	Cohort	8	very low	NS
Bladder cancer	SSB	Li 2021	Cohort, case-control and/or cross-sectional	8	very low	NS
Breast cancer	SSB	Llaha 2021	Cohort, case-control and/or cross-sectional	7	very low	IV
Breast cancer mortality	SSB	Li 2021	Cohort	8	very low	IV
Colon cancer	SSB	Zhang 2010	Cohort	4	very low	NS
Colorectal cancer	SSB	Li 2021	Cohort	8	very low	NS
Colorectal cancer	Added sugars	Galeone 2012	Cohort	3	very low	NS
Colorectal cancer mortality	SSB	Li 2021	Cohort	8	very low	NS
Endometrial cancer	SSB	Li 2021	Cohort	8	very low	NS
Esophageal cancer	SSB	Li 2021	Cohort, case-control and/or cross-sectional	8	very low	NS

Gastric cancer	SSB	Li 2021	Cohort	8	very low	NS
Glioma	SSB	Li 2021	Cohort	8	very low	IV
Hematologic malignancy	SSB	Li 2021	Cohort	8	very low	NS
Kidney cancer	SSB	Li 2021	Cohort	8	very low	NS
Lung cancer mortality	SSB	Li 2021	Cohort	8	very low	NS
Nasopharyngeal carcinoma	SSB	Li 2021	Cohort, case-control and/or cross-sectional	8	very low	NS
Ovarian cancer	Lactose	Liu 2015	Cohort	5	very low	NS
Overall cancer mortality	SSB	Li 2021	Cohort	8	very low	IV
Overall cancer risk	SSB	Li 2021	Cohort	8	very low	IV
Pancreatic cancer	SSB	Li 2021	Cohort	8	very low	NS
Pancreatic cancer	Sucrose	Aune 2012	Cohort	6	very low	NS
Prostate cancer	SSB	Li 2021	Cohort	8	very low	III
Prostate cancer mortality	SSB	Li 2021	Cohort	8	very low	NS
Other outcomes						
Depression	SSB	Hu 2019	Cohort, case-control and/or cross-sectional	9	low	II
All-cause mortality	SSB	Pan 2021	Cohort	10	low	III
ALT	Fructose	Chiu 2014	RCT	9	low	NS
ADHD	SSB	Farsad-Naeimi 2020	Case-control and/or cross-sectional	7	very low	IV
ADHD	Total sugars	Farsad-Naeimi 2020	Cohort, case-control and/or cross-sectional	7	very low	IV
Asthma in offspring[c]	Free sugars	Gupta 2021	Cohort	8	very low	NS
Asthma[a]	SSB	Al-Zalabani 2019	Cohort, case-control and/or cross-sectional	8	very low	IV

BMD	SSB	Ahn 2021	Case-control and/or cross-sectional	8	very low	IV
CKD	SSB	Lo 2021	Cohort, case-control and/or cross-sectional	9	very low	NS
Dental caries	SSB	Valenzuela 2021	Cohort, case-control and/or cross-sectional	10	very low	II
Dental erosion	SSB	Valenzuela 2021	Case-control and/or cross-sectional	10	very low	III
IHCL	Fructose	Chiu 2014	RCT	9	very low	IV
NAFLD	SSB	Chen 2019	Cohort, case-control and/or cross-sectional	8	very low	II

AMSTAR, a measurement tool to assess systematic reviews; GRADE, Grading of Recommendations Assessment, Development, and Evaluation; Δ, final value − baseline value; SSB, sugar-sweetened beverage; RCT, randomized controlled trial; NS, non-significant; BMI, body mass index; HDL-C, high-density lipoprotein cholesterol; LADA, latent autoimmune diabetes in adults; LDL-C, low-density lipoprotein cholesterol; TG, triglycerides; T2DM, type 2 diabetes mellitus; TC, total cholesterol; CHD, coronary heart disease; MI, myocardial infarction; CVD, cardiovascular disease; DBP, diastolic blood pressure; SBP, systolic blood pressure; ALT, alanine aminotransferase; ADHD, Attention Deficit Hyperactivity Disorder; BMD, bone mineral density; CKD, chronic kidney disease; IHCL, intrahepatocellular lipids; NAFLD, non-alcoholic fatty liver disease.

[a] Children.

[b] Children and adolescence.

[c] Maternal increased free sugar intake during pregnancy.

Supplementary Table F. Assessments of AMSTAR scores.

Outcome	Category	Assessed with	Author	Year	A priori design provided	Duplicate study selection & data extraction	At least two electronic databases searched	Status of publication used as an inclusion criterion	List of included and excluded studies provided	Characteristics of included studies provided	Scientific quality of included studies assessed	Scientific quality of the included studies used appropriately to form conclusions	Appropriate methods to combine studies	Publication bias assessed	Conflict of interest included	Total AMSTAR Score
ADHD	SSB	Highest versus lowest	Farsad-Naeimi	2020	0	1	1	0	1	1	1	0	1	0	1	7
ADHD	Total sugars	Highest versus lowest	Farsad-Naeimi	2020	0	1	1	0	1	1	1	0	1	0	1	7
All-cause mortality	SSB	250 mL/d increment	Pan	2021	1	1	1	0	1	1	1	1	1	1	1	10
ALT	Fructose	Any versus none	Chiu	2014	1	1	1	0	1	1	1	0	1	1	1	9
Asthma in offspring[a]	Free sugars	Highest versus lowest	Gupta	2021	1	1	1	0	1	1	1	0	1	0	1	8
Asthma[b]	SSB	Highest versus lowest	Al-Zalabani	2019	0	1	1	0	1	1	1	0	1	1	1	8
Biliary track cancer	SSB	Highest versus lowest	Li	2021	0	1	1	0	1	1	1	0	1	1	1	8
Bladder cancer	SSB	Highest versus lowest	Li	2021	0	1	1	0	1	1	1	0	1	1	1	8
BMD	SSB	Highest versus lowest	Ahn	2021	1	1	1	0	1	1	1	0	1	0	1	8
Breast cancer	SSB	Highest versus lowest	Llaha	2021	0	1	1	0	1	1	1	0	1	0	1	7
Breast cancer mortality	SSB	Highest versus lowest	Li	2021	0	1	1	0	1	1	1	0	1	1	1	8
CHD	SSB	250 mL/d increment	Bechthold	2017	1	1	1	0	1	1	0	1	1	1	1	9
CKD	SSB	Highest versus lowest	Lo	2021	1	1	1	0	1	1	1	0	1	1	1	9
Colon cancer	SSB	375 g/d increment	Zhang	2010	1	0	0	0	0	1	0	0	1	0	1	4
Colorectal cancer	SSB	Highest versus lowest	Li	2021	0	1	1	0	1	1	1	0	1	1	1	8
Colorectal cancer	Added sugars	Highest versus lowest	Galeone	2012	0	0	0	0	0	0	0	0	1	1	1	3
Colorectal cancer mortality	SSB	Highest versus lowest	Li	2021	0	1	1	0	1	1	1	0	1	1	1	8
CVD	SSB	1 serving/d increment	Yin	2021	1	1	1	0	1	1	1	0	1	1	1	9
CVD	Sucrose	Highest versus lowest	Khan	2019	1	1	1	0	1	1	1	1	1	1	1	10
CVD	Total sugars	Highest versus lowest	Khan	2019	1	1	1	0	1	1	1	1	1	1	1	10
CVD mortality	SSB	1 serving/d increment	Zhang	2021	0	1	1	1	1	1	1	1	1	1	1	10
CVD mortality	Fructose	Highest versus lowest	Khan	2019	1	1	1	0	1	1	1	1	1	1	1	10
CVD mortality	Sucrose	Highest versus lowest	Khan	2019	1	1	1	0	1	1	1	1	1	1	1	10
CVD mortality	Total sugars	Highest versus lowest	Khan	2019	1	1	1	0	1	1	1	1	1	1	1	10
CVD mortality	Added sugars	Highest versus lowest	Khan	2019	1	1	1	0	1	1	1	1	1	1	1	10
Dental caries	SSB	Never/low versus moderate/high consumption	Valenzuela	2021	1	1	1	0	1	1	1	1	1	1	1	10
Dental erosion	SSB	Never/low versus moderate/high consumption	Valenzuela	2021	1	1	1	0	1	1	1	1	1	1	1	10
Depression	SSB	Highest versus lowest	Hu	2019	0	1	1	1	1	1	1	0	1	1	1	9
Endometrial cancer	SSB	Highest versus lowest	Li	2021	0	1	1	0	1	1	1	0	1	1	1	8
Esophageal cancer	SSB	Highest versus lowest	Li	2021	0	1	1	0	1	1	1	0	1	1	1	8
Gastric cancer	SSB	Highest versus lowest	Li	2021	0	1	1	0	1	1	1	0	1	1	1	8
Glioma	SSB	Highest versus lowest	Li	2021	0	1	1	0	1	1	1	0	1	1	1	8
Gout	SSB	Highest versus lowest	Ebrahimpour-koujan	2020	0	1	1	1	1	1	1	0	1	1	1	9
Gout (dose-response)	SSB	1 serving/week increment	Ayoub-Charette	2019	1	1	1	0	1	1	1	1	1	0	1	9
Gout	Fructose	Highest versus lowest	Jamnik	2016	1	1	1	0	1	1	1	1	1	0	1	9
HDL-C	SSB	Highest versus lowest	Nikniaz	2021	1	1	1	0	1	1	1	1	1	1	1	10
Heart failure	SSB	Highest versus lowest	Bechthold	2017	1	1	1	0	1	1	0	1	1	1	1	9
Hematologic malignancy	SSB	Highest versus lowest	Li	2021	0	1	1	0	1	1	1	0	1	1	1	8
Hepatocellular carcinoma	SSB	Highest versus lowest	Li	2021	0	1	1	0	1	1	1	0	1	1	1	8
Hypertension	SSB	Highest versus lowest	Liu	2019	1	1	1	1	1	1	1	1	1	1	1	11
Hypertension	Fructose	Highest versus lowest	Jayalath	2014	1	1	1	0	1	1	1	0	1	1	1	9
Hypertension[c]	SSB	Highest versus lowest	Farhangi	2020	0	1	1	0	1	1	1	0	1	1	1	8
Hyperuricemia	SSB	Highest versus lowest	Ebrahimpour-koujan	2020	0	1	1	1	1	1	1	0	1	1	1	9
Hyperuricemia	Fructose	Highest versus lowest	Li	2018	0	1	1	0	1	1	1	0	1	1	1	8
IHCL	Fructose	Any versus none	Chiu	2014	1	1	1	0	1	1	1	0	1	1	1	9

Kidney cancer	SSB	Highest versus lowest	Li	2021	0	1	1	0	1	1	1	0	1	1	1	8
LADA	SSB	Highest versus lowest	El-Malky	2020	0	1	1	1	0	1	0	0	1	1	1	7
Large waist circumference	SSB	Highest versus lowest	Ardeshirlarijani	2021	1	1	1	0	1	1	1	0	1	1	1	9
LDL-C	SSB	Highest versus lowest	Nikniaz	2021	1	1	1	0	1	1	1	1	1	1	1	10
Liver fat accumulation	Added sugars	Any versus none	Ma	2016	0	1	1	0	1	1	1	0	1	1	1	8
Lung cancer mortality	SSB	Highest versus lowest	Li	2021	0	1	1	0	1	1	1	0	1	1	1	8
Metabolic syndrome	SSB	355 mL/d increment	Semnani-Azad	2020	0	1	1	1	1	1	1	1	1	1	1	10
MI	SSB	Highest versus lowest	Narain	2016	0	1	1	0	1	1	1	0	1	1	1	8
Muscle fat accumulation	Added sugars	Any versus none	Ma	2016	0	1	1	0	1	1	1	0	1	1	1	8
NAFLD	SSB	Highest versus lowest	Chen	2019	0	1	1	0	1	1	1	0	1	1	1	8
Nasopharyngeal carcinoma	SSB	Highest versus lowest	Li	2021	0	1	1	0	1	1	1	0	1	1	1	8
Obesity	SSB	250 mL/d increment	Qin	2020	0	1	1	0	1	1	1	0	1	1	1	8
Obesity[b]	SSB	Any versus none	Morenga	2013	0	1	1	0	1	1	1	1	1	1	1	9
Ovarian cancer	Lactose	Highest versus lowest	Liu	2015	0	0	1	0	0	1	0	0	1	1	1	5
Overall cancer mortality	SSB	Highest versus lowest	Li	2021	0	1	1	0	1	1	1	0	1	1	1	8
Overall cancer risk	SSB	1 serving/d increment	Li	2021	0	1	1	0	1	1	1	0	1	1	1	8
Pancreatic cancer	Fructose	25 g/d increment	Aune	2012	0	1	1	0	0	1	0	0	1	1	1	6
Pancreatic cancer	SSB	Highest versus lowest	Li	2021	0	1	1	0	1	1	1	0	1	1	1	8
Pancreatic cancer	Sucrose	25 g/d increment	Aune	2012	0	1	1	0	0	1	0	0	1	1	1	6
Postprandial TG	Fructose	Any versus none	Wang	2014	1	1	1	0	1	1	1	0	1	1	1	9
Prostate cancer	SSB	Highest versus lowest	Li	2021	0	1	1	0	1	1	1	0	1	1	1	8
Prostate cancer mortality	SSB	Highest versus lowest	Li	2021	0	1	1	0	1	1	1	0	1	1	1	8
Serum uric acid	SSB	Highest versus lowest	Ebrahimpour-koujan	2021	1	1	1	1	1	1	1	0	1	1	1	10
Serum uric acid	Fructose	Any versus none	Wang	2012	1	1	1	0	1	1	1	0	1	1	1	9
Stroke	SSB	250 mL/d increment	Bechthold	2017	1	1	1	0	1	1	0	1	1	1	1	9
T2DM	SSB	1 serving/d increment	Meng	2021	0	1	1	0	1	1	1	0	1	1	1	8
T2DM	Sucrose	25 g/d increment	Tsilas	2017	1	1	1	0	1	1	1	1	1	1	1	10
T2DM	Fructose	Highest versus lowest	Tsilas	2017	1	1	1	0	1	1	1	1	1	1	1	10
T2DM	Total sugars	Highest versus lowest	Tsilas	2017	1	1	1	0	1	1	1	1	1	1	1	10
TC	SSB	Highest versus lowest	Nikniaz	2021	1	1	1	0	1	1	1	1	1	1	1	10
TG	SSB	Highest versus lowest	Nikniaz	2021	1	1	1	0	1	1	1	1	1	1	1	10
Δ BMI	SSB	Reducing SSB consumption versus not reducing	Mattes	2011	0	1	1	1	1	1	1	0	1	0	1	8
Δ BMI[b]	SSB	1 serving/d increment	Malik	2013	0	1	1	0	1	1	1	0	1	1	1	8
Δ Body weight	SSB	Highest versus lowest	Malik	2013	0	1	1	0	1	1	1	0	1	1	1	8
Δ Body weight	Fructose	Any versus none	Sievenpiper	2012	0	1	1	0	1	1	1	0	1	1	1	8
Δ Body weight (one year)	SSB	1 serving/d increment	Malik	2013	0	1	1	0	1	1	1	0	1	1	1	8
Δ DBP[c]	SSB	Highest versus lowest	Farhangi	2020	0	1	1	0	1	1	1	0	1	1	1	8
Δ SBP[c]	SSB	Highest versus lowest	Farhangi	2020	0	1	1	0	1	1	1	0	1	1	1	8

AMSTAR, a measurement tool to assess systematic reviews; ADHD, Attention Deficit Hyperactivity Disorder; SSB, sugar-sweetened beverage; ALT, alanine aminotransferase; BMD, bone mineral density; CHD, coronary heart disease; CKD, chronic kidney disease; CVD, cardiovascular disease; HDL-C, high-density lipoprotein cholesterol; IHCL, intrahepatocellular lipids; LADA, latent autoimmune diabetes in adults; LDL-C, low-density lipoprotein cholesterol; MI, myocardial infarction; NAFLD, non-alcoholic fatty liver disease; TG, triglycerides; T2DM, type 2 diabetes mellitus; TC, total cholesterol; Δ, final value − baseline value; BMI, body mass index; DBP, diastolic blood pressure; SBP, systolic blood pressure.

[a] Maternal increased free sugar intake during pregnancy.

[b] Children.

[c] Children and adolescence.

Supplementary Table G. GRADE classification of quality of evidence.

Outcome	Category	Assessed with	Author	Year	No. of studies	RCT	Cohort	Case-control/ cross-sectional	Risk of bias	Inconsistency	Indirectness	Imprecision	Publication bias	Plausible confounding	Magnitude of effect	Dose-response gradient	Quality
ADHD	SSB	Highest versus lowest	Farsad-Naeimi	2020	2	0	0	2	serious risk	no serious inconsistency	no serious indirectness	no serious imprecision	NA	would not reduce effect	no	no	very low
ADHD	Total sugars	Highest versus lowest	Farsad-Naeimi	2020	7	0	3	4	serious risk	serious inconsistency	serious indirectness	no serious imprecision	NA	would not reduce effect	no	no	very low
All-cause mortality	SSB	250 mL/d increment	Pan	2021	11	0	11	0	no serious risk	serious inconsistency	no serious indirectness	no serious imprecision	undetected	would not reduce effect	no	yes	low
ALT	Fructose	Any versus none	Chiu	2014	6	6	0	0	no serious risk	no serious inconsistency	serious indirectness	serious imprecision	undetected	would not reduce effect	no	no	low
Asthma in offspring[a]	Free sugars	Highest versus lowest	Gupta	2021	2	0	2	0	serious risk	no serious inconsistency	serious indirectness	no serious imprecision	NA	would not reduce effect	no	no	very low
Asthma[b]	SSB	Highest versus lowest	Al-Zalabani	2019	7	0	1	6	serious risk	serious inconsistency	no serious indirectness	serious imprecision	undetected	would not reduce effect	no	no	very low
Biliary track cancer	SSB	Highest versus lowest	Li	2021	2	0	2	0	serious risk	serious inconsistency	no serious indirectness	no serious imprecision	NA	would not reduce effect	no	no	very low
Bladder cancer	SSB	Highest versus lowest	Li	2021	6	0	1	5	serious risk	no serious inconsistency	no serious indirectness	serious imprecision	NA	would not reduce effect	no	no	very low
BMD	SSB	Highest versus lowest	Ahn	2021	10	0	0	10	serious risk	serious inconsistency	no serious indirectness	no serious imprecision	NA	would reduce effect	no	no	very low
Breast cancer	SSB	Highest versus lowest	Llaha	2021	6	0	4	2	serious risk	no serious inconsistency	no serious indirectness	serious imprecision	NA	would not reduce effect	no	no	very low
Breast cancer mortality	SSB	Highest versus lowest	Li	2021	2	0	2	0	serious risk	no serious inconsistency	no serious indirectness	no serious imprecision	NA	would not reduce effect	no	no	very low
CHD	SSB	250 mL/d increment	Bechthold	2017	4	0	4	0	serious risk	no serious inconsistency	no serious indirectness	no serious imprecision	NA	would not reduce effect	no	yes	low
CKD	SSB	Highest versus lowest	Lo	2021	6	0	5	1	serious risk	serious inconsistency	no serious indirectness	serious imprecision	undetected	would not reduce effect	no	no	very low
Colon cancer	SSB	375 g/d increment	Zhang	2010	13	0	13	0	serious risk	no serious inconsistency	no serious indirectness	no serious imprecision	NA	would not reduce effect	no	no	very low
Colorectal cancer	SSB	Highest versus lowest	Li	2021	6	0	6	0	serious risk	no serious inconsistency	no serious indirectness	serious imprecision	NA	would not reduce effect	no	no	very low
Colorectal cancer	Added sugars	Highest versus lowest	Galeone	2012	2	0	2	0	serious risk	no serious inconsistency	serious indirectness	no serious imprecision	NA	would not reduce effect	no	no	very low
Colorectal cancer mortality	SSB	Highest versus lowest	Li	2021	6	0	6	0	serious risk	serious inconsistency	no serious indirectness	serious imprecision	NA	would not reduce effect	no	no	very low
CVD	SSB	1 serving/d increment	Yin	2021	7	0	7	7	no serious risk	serious inconsistency	no serious indirectness	serious imprecision	undetected	would not reduce effect	no	yes	very low
CVD	Sucrose	Highest versus lowest	Khan	2019	3	0	3	0	no serious risk	no serious inconsistency	serious indirectness	no serious imprecision	NA	would not reduce effect	no	no	very low
CVD	Total sugars	Highest versus lowest	Khan	2019	8	0	8	0	no serious risk	no serious inconsistency	serious indirectness	no serious imprecision	NA	would not reduce effect	no	no	very low
CVD mortality	SSB	1 serving/d increment	Zhang	2021	13	0	13	0	no serious risk	no serious inconsistency	no serious indirectness	serious imprecision	strongly suspected	would not reduce effect	no	yes	very low
CVD mortality	Fructose	Highest versus lowest	Khan	2019	2	0	2	0	no serious risk	no serious inconsistency	serious indirectness	serious imprecision	NA	would not reduce effect	no	yes	very low
CVD mortality	Sucrose	Highest versus lowest	Khan	2019	2	0	2	0	no serious risk	no serious inconsistency	serious indirectness	serious imprecision	NA	would not reduce effect	no	yes	very low
CVD mortality	Total sugars	Highest versus lowest	Khan	2019	4	0	4	0	no serious risk	no serious inconsistency	serious indirectness	serious imprecision	NA	would not reduce effect	no	yes	very low
CVD mortality	Added sugars	Highest versus lowest	Khan	2019	4	0	4	0	no serious risk	serious inconsistency	serious indirectness	serious imprecision	NA	would not reduce effect	no	yes	very low
Dental caries	SSB	Never/low versus	Valenzuela	2021	17	0	1	16	serious risk	serious	no serious	no serious	undetected	would not	no	yes	very low

		moderate/high consumption								inconsistency	indirectness	imprecision		reduce effect			
Dental erosion	SSB	Never/low versus moderate/high consumption	Valenzuela	2021	13	0	0	13	no serious risk	serious inconsistency	no serious indirectness	no serious imprecision	undetected	would not reduce effect	no	no	very low
Depression	SSB	Highest versus lowest	Hu	2019	10	0	4	6	serious risk	no serious inconsistency	no serious indirectness	no serious imprecision	undetected	would not reduce effect	no	yes	low
Endometrial cancer	SSB	Highest versus lowest	Li	2021	2	0	2	0	serious risk	serious inconsistency	no serious indirectness	serious imprecision	NA	would not reduce effect	no	no	very low
Esophageal cancer	SSB	Highest versus lowest	Li	2021	5	0	1	4	serious risk	serious inconsistency	no serious indirectness	serious imprecision	NA	would not reduce effect	no	no	very low
Gastric cancer	SSB	Highest versus lowest	Li	2021	2	0	2	0	serious risk	no serious inconsistency	no serious indirectness	serious imprecision	NA	would not reduce effect	no	no	very low
Glioma	SSB	Highest versus lowest	Li	2021	2	0	2	0	serious risk	no serious inconsistency	no serious indirectness	serious imprecision	NA	would not reduce effect	no	no	very low
Gout	SSB	Highest versus lowest	Ebrahimpour-koujan	2020	3	0	3	0	serious risk	no serious inconsistency	no serious indirectness	no serious imprecision	undetected	would not reduce effect	no	no	very low
Gout (dose-response)	SSB	1 serving/week increment	Ayoub-Charette	2019	2	0	2	0	no serious risk	no serious inconsistency	serious indirectness	no serious imprecision	undetected	would not reduce effect	no	yes	low
Gout	Fructose	Highest versus lowest	Jamnik	2016	2	0	2	0	no serious risk	no serious inconsistency	serious indirectness	no serious imprecision	NA	would not reduce effect	no	no	very low
HDL-C	SSB	Highest versus lowest	Nikniaz	2021	14	0	1	13	serious risk	serious inconsistency	no serious indirectness	serious imprecision	undetected	would not reduce effect	no	no	very low
Heart failure	SSB	Highest versus lowest	Bechthold	2017	2	0	2	0	serious risk	serious inconsistency	no serious indirectness	serious imprecision	NA	would not reduce effect	no	yes	very low
Hematologic malignancy	SSB	Highest versus lowest	Li	2021	2	0	2	0	serious risk	no serious inconsistency	no serious indirectness	serious imprecision	NA	would not reduce effect	no	no	very low
Hepatocellular carcinoma	SSB	Highest versus lowest	Li	2021	2	0	1	1	serious risk	no serious inconsistency	no serious indirectness	no serious imprecision	NA	would not reduce effect	yes	no	low
Hypertension	SSB	Highest versus lowest	Liu	2019	13	0	13	0	no serious risk	serious inconsistency	no serious indirectness	serious imprecision	strongly suspected	would not reduce effect	no	yes	very low
Hypertension	Fructose	Highest versus lowest	Jayalath	2014	3	0	3	0	no serious risk	no serious inconsistency	serious indirectness	no serious imprecision	undetected	would not reduce effect	no	yes	low
Hypertension[c]	SSB	Highest versus lowest	Farhangi	2020	5	0	0	5	no serious risk	no serious inconsistency	no serious indirectness	serious imprecision	undetected	would not reduce effect	no	no	very low
Hyperuricemia	SSB	Highest versus lowest	Ebrahimpour-koujan	2020	6	0	0	6	serious risk	no serious inconsistency	no serious indirectness	no serious imprecision	undetected	would not reduce effect	no	no	very low
Hyperuricemia	Fructose	Highest versus lowest	Li	2018	4	0	0	4	no serious risk	no serious inconsistency	serious indirectness	no serious imprecision	undetected	would not reduce effect	no	no	very low
IHCL	Fructose	Any versus none	Chiu	2014	5	5	0	0	serious risk	serious inconsistency	no serious indirectness	serious imprecision	undetected	would not reduce effect	no	no	very low
Kidney cancer	SSB	Highest versus lowest	Li	2021	2	0	2	0	serious risk	no serious inconsistency	no serious indirectness	serious imprecision	NA	would not reduce effect	no	no	very low
LADA	SSB	Highest versus lowest	El-Malky	2020	6	0	0	6	serious risk	serious inconsistency	no serious indirectness	serious imprecision	undetected	would not reduce effect	no	no	very low
Large waist circumference	SSB	Highest versus lowest	Ardeshirlarijani	2021	10	0	10	0	no serious risk	serious inconsistency	no serious indirectness	serious imprecision	NA	would not reduce effect	no	no	very low
LDL-C	SSB	Highest versus lowest	Nikniaz	2021	9	0	1	8	serious risk	serious inconsistency	no serious indirectness	no serious imprecision	undetected	would not reduce effect	no	yes	very low
Liver fat accumulation	Added sugars	Any versus none	Ma	2016	8	8	0	0	no serious risk	no serious inconsistency	no serious indirectness	serious imprecision	undetected	would not reduce effect	no	no	moderate
Lung cancer mortality	SSB	Highest versus lowest	Li	2021	2	0	2	0	serious risk	no serious inconsistency	no serious indirectness	no serious imprecision	NA	would not reduce effect	no	no	very low
Metabolic syndrome	SSB	355 mL/d increment	Semnani-Azad	2020	6	0	6	0	no serious risk	serious inconsistency	no serious indirectness	serious imprecision	NA	would not reduce effect	no	yes	very low
MI	SSB	Highest versus lowest	Narain	2016	4	0	4	0	no serious	no serious	no serious	no serious	NA	would not	no	no	low

									risk	inconsistency	indirectness	imprecision		reduce effect			
Muscle fat accumulation	Added sugars	Any versus none	Ma	2016	5	5	0	0	no serious risk	no serious inconsistency	no serious indirectness	serious imprecision	undetected	would not reduce effect	no	no	moderate
NAFLD	SSB	Highest versus lowest	Chen	2019	12	0	1	11	serious risk	serious inconsistency	no serious indirectness	no serious imprecision	strongly suspected	would not reduce effect	no	yes	very low
Nasopharyngeal carcinoma	SSB	Highest versus lowest	Li	2021	2	0	1	1	serious risk	no serious inconsistency	no serious indirectness	serious imprecision	NA	would not reduce effect	no	no	very low
Obesity	SSB	250 mL/d increment	Qin	2020	7	0	7	0	no serious risk	serious inconsistency	no serious indirectness	serious imprecision	strongly suspected	would not reduce effect	no	yes	very low
Obesity[b]	SSB	Any versus none	Morenga	2013	7	0	7	0	no serious risk	no serious inconsistency	no serious indirectness	no serious imprecision	NA	would not reduce effect	no	no	low
Ovarian cancer	Lactose	Highest versus lowest	Liu	2015	14	0	14	0	serious risk	no serious inconsistency	serious indirectness	serious imprecision	undetected	would not reduce effect	no	no	very low
Overall cancer mortality	SSB	Highest versus lowest	Li	2021	10	0	10	0	serious risk	serious inconsistency	no serious indirectness	no serious imprecision	undetected	would not reduce effect	no	no	very low
Overall cancer risk	SSB	1 serving/d increment	Li	2021	20	0	20	0	serious risk	serious inconsistency	no serious indirectness	no serious imprecision	strongly suspected	would not reduce effect	no	yes	very low
Pancreatic cancer	Fructose	25 g/d increment	Aune	2012	6	0	6	0	serious risk	no serious inconsistency	serious indirectness	no serious imprecision	undetected	would reduce effect	no	yes	low
Pancreatic cancer	SSB	Highest versus lowest	Li	2021	6	0	6	0	serious risk	serious inconsistency	no serious indirectness	serious imprecision	NA	would not reduce effect	no	no	very low
Pancreatic cancer	Sucrose	25 g/d increment	Aune	2012	8	0	8	0	serious risk	serious inconsistency	serious indirectness	no serious imprecision	undetected	would not reduce effect	no	no	very low
Postprandial TG	Fructose	Any versus none	Wang	2014	5	5	0	0	serious risk	serious inconsistency	no serious indirectness	serious imprecision	undetected	would not reduce effect	no	no	very low
Prostate cancer	SSB	Highest versus lowest	Li	2021	6	0	6	0	serious risk	no serious inconsistency	no serious indirectness	serious imprecision	NA	would not reduce effect	no	no	very low
Prostate cancer mortality	SSB	Highest versus lowest	Li	2021	2	0	2	0	serious risk	no serious inconsistency	no serious indirectness	no serious imprecision	NA	would not reduce effect	no	no	very low
Serum uric acid	SSB	Highest versus lowest	Ebrahimpour-koujan	2021	6	0	0	6	serious risk	no serious inconsistency	no serious indirectness	no serious imprecision	undetected	would not reduce effect	no	no	very low
Serum uric acid	Fructose	Any versus none	Wang	2012	9	9	0	0	no serious risk	no serious inconsistency	no serious indirectness	serious imprecision	undetected	would not reduce effect	no	no	moderate
Stroke	SSB	250 mL/d increment	Bechthold	2017	6	0	6	0	serious risk	no serious inconsistency	no serious indirectness	serious imprecision	NA	would not reduce effect	no	yes	very low
T2DM	SSB	1 serving/d increment	Meng	2021	16	0	16	0	no serious risk	serious inconsistency	no serious indirectness	no serious imprecision	strongly suspected	would not reduce effect	no	yes	very low
T2DM	Sucrose	25 g/d increment	Tsilas	2017	6	0	6	0	no serious risk	no serious inconsistency	serious indirectness	serious imprecision	NA	would not reduce effect	no	yes	very low
T2DM	Fructose	Highest versus lowest	Tsilas	2017	6	0	6	0	no serious risk	serious inconsistency	serious indirectness	serious imprecision	NA	would not reduce effect	no	no	very low
T2DM	Total sugars	Highest versus lowest	Tsilas	2017	12	0	12	0	no serious risk	serious inconsistency	serious indirectness	no serious imprecision	undetected	would not reduce effect	no	no	very low
TC	SSB	Highest versus lowest	Nikniaz	2021	7	0	0	7	serious risk	serious inconsistency	no serious indirectness	no serious imprecision	undetected	would not reduce effect	no	yes	very low
TG	SSB	Highest versus lowest	Nikniaz	2021	13	0	1	12	serious risk	serious inconsistency	no serious indirectness	no serious imprecision	undetected	would not reduce effect	no	no	very low
Δ BMI	SSB	Reducing SSB consumption versus not reducing	Mattes	2011	6	6	0	0	serious risk	no serious inconsistency	no serious indirectness	serious imprecision	NA	would not reduce effect	no	no	very low
Δ BMI[b]	SSB	1 serving/d increment	Malik	2013	20	0	20	0	no serious risk	serious inconsistency	no serious indirectness	no serious imprecision	undetected	would not reduce effect	no	yes	low
Δ Body weight	SSB	Highest versus lowest	Malik	2013	6	6	0	0	no serious risk	no serious inconsistency	no serious indirectness	serious imprecision	undetected	would not reduce effect	no	no	moderate
Δ Body weight	Fructose	Any versus none	Sievenpiper	2012	13	13	0	0	serious risk	no serious	no serious	serious	undetected	would not	no	no	low

										inconsistency	indirectness	imprecision		reduce effect			
Δ Body weight (one year)	SSB	1 serving/d increment	Malik	2013	8	0	8	0	no serious risk	serious inconsistency	no serious indirectness	no serious imprecision	strongly suspected	would not reduce effect	no	yes	very low
Δ DBP[c]	SSB	Highest versus lowest	Farhangi	2020	14	0	4	10	serious risk	serious inconsistency	no serious indirectness	no serious imprecision	undetected	would not reduce effect	no	no	very low
Δ SBP[c]	SSB	Highest versus lowest	Farhangi	2020	15	0	4	11	serious risk	serious inconsistency	no serious indirectness	no serious imprecision	undetected	would not reduce effect	no	no	very low

GRADE, Grading of Recommendations Assessment, Development, and Evaluation; RCT, randomized controlled trial; ADHD, Attention Deficit Hyperactivity Disorder; SSB, sugar-sweetened beverage; NA, not available; ALT, alanine aminotransferase; BMD, bone mineral density; CHD, coronary heart disease; CKD, chronic kidney disease; CVD, cardiovascular disease; HDL-C, high-density lipoprotein cholesterol; IHCL, intrahepatocellular lipids; LADA, latent autoimmune diabetes in adults; LDL-C, low-density lipoprotein cholesterol; MI, myocardial infarction; NAFLD, non-alcoholic fatty liver disease; TG, triglycerides; T2DM, type 2 diabetes mellitus; TC, total cholesterol; Δ, final value − baseline value; BMI, body mass index; DBP, diastolic blood pressure; SBP, systolic blood pressure.

[a] Maternal increased free sugar intake during pregnancy.

[b] Children.

[c] Children and adolescence.
